# Antibody bivalency improves antiviral efficacy by inhibiting virion release independently of Fc gamma receptors

**DOI:** 10.1016/j.celrep.2022.110303

**Published:** 2022-02-01

**Authors:** Mehmet Sahin, Melissa M. Remy, Benedict Fallet, Rami Sommerstein, Marianna Florova, Anna Langner, Katja Klausz, Tobias Straub, Mario Kreutzfeldt, Ingrid Wagner, Cinzia T. Schmidt, Pauline Malinge, Giovanni Magistrelli, Shozo Izui, Hanspeter Pircher, J. Sjef Verbeek, Doron Merkler, Matthias Peipp, Daniel D. Pinschewer

**Affiliations:** 1 Department of Biomedicine – Haus Petersplatz, Division of Experimental Virology, University of Basel, 4009 Basel, Switzerland; 2 Department of Pathology and Immunology, University of Geneva, 1211 Geneva, Switzerland; 3 Division of Stem Cell Transplantation and Immunotherapy, Department of Medicine II, University Hospital Schleswig-Holstein and Christian-Albrechts-University Kiel, Kiel, Germany; 4 Institute for Immunology, Department for Medical Microbiology and Hygiene, University Medical Center Freiburg, 79104 Freiburg, Germany; 5 Department of Pathology and Immunology, Division of Clinical Pathology, University and University Hospital of Geneva, 1211 Geneva, Switzerland; 6 BioEM Lab, Center for Cellular Imaging & Nano Analytics, Biozentrum, University of Basel, Basel, Switzerland; 7 Light Chain Bioscience, Novimmune SA, Plan-les-Ouates, Switzerland; 8 Department of Human Genetics, Leiden University Medical Center, Leiden, the Netherlands; 9 Department of Biomedical Engineering, Toin University of Yokohama, Yokohama, Japan

**Keywords:** antibody bivalency, immunoglobulin superfamily, antiviral protection, virus neutralization, lymphocytic choriomeningitis virus (LCMV), inhibition of virion release, Fc gamma receptors, virus budding, humoral immunity

## Abstract

Across the animal kingdom, multivalency discriminates antibodies from all other immunoglobulin superfamily members. The evolutionary forces conserving multivalency above other structural hallmarks of antibodies remain, however, incompletely defined.

Here, we engineer monovalent either Fc-competent or -deficient antibody formats to investigate mechanisms of protection of neutralizing antibodies (nAbs) and non-neutralizing antibodies (nnAbs) in virus-infected mice. Antibody bivalency enables the tethering of virions to the infected cell surface, inhibits the release of virions in cell culture, and suppresses viral loads *in vivo* independently of Fc gamma receptor (FcγR) interactions. In return, monovalent antibody formats either do not inhibit virion release and fail to protect *in vivo* or their protective efficacy is largely FcγR dependent. Protection in mice correlates with virus-release-inhibiting activity of nAb and nnAb rather than with their neutralizing capacity.

These observations provide mechanistic insights into the evolutionary conservation of antibody bivalency and help refining correlates of nnAb protection for vaccine development.

## Highlights

• Non-neutralizing antibodies inhibit virus release from cells to control infection
• Antibody bivalency enables virus release inhibition independently of Fc receptors
• Bivalent but not monovalent antibodies protect mice independently of Fc receptors

## Graphical abstract

**Figure:**
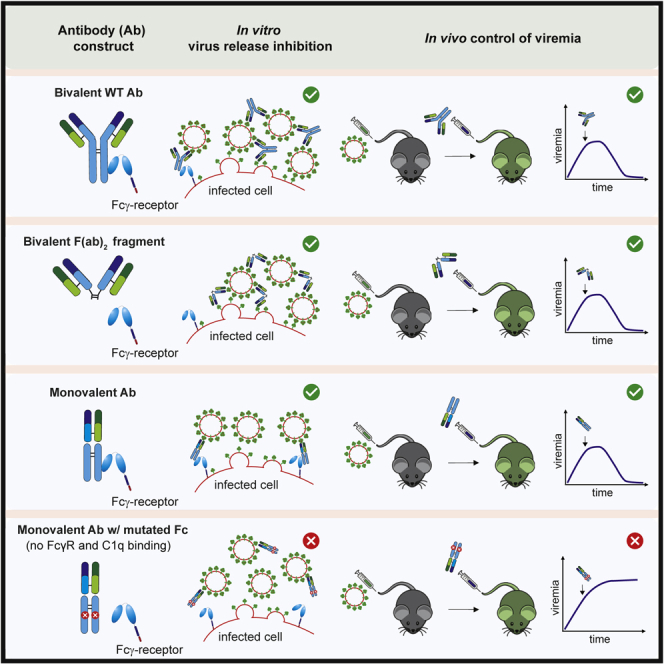

## Teaser

The evolutionary conservation of antibody bivalency remains incompletely understood. Sahin et al. demonstrate that antibody-mediated inhibition of virion release is key to *in vivo* protection. Moreover, they find bivalent but not engineered monovalent antibodies inhibit virion release independently of Fc gamma receptors and control virus infection in Fc gamma receptor-deficient mice.

## Introduction

Antibody responses constitute a key element of antiviral adaptive immunity and, with the recent advancements in the field of molecular engineering, antibodies have also become a key therapeutic modality for a wide array of diseases (Winter, 2019). These include viral infections of global significance, such as severe acute respiratory syndrome coronavirus-2 (SARS-CoV-2) and respiratory syncytial virus (Abraham, 2020; Cao et al., 2020; Englund, 1999; Soto et al., 2020), rendering it important to better understand how antiviral antibodies protect *in vivo*.

Antibodies belong to the immunoglobulin superfamily, a large class of cell surface molecules and soluble proteins that are involved in binding, adhesion, and recognition processes of cells, the latter prominently represented by the hypervariable T cell and B cell receptors. B cell receptors are secreted as soluble immunoglobulins or antibodies upon B cell differentiation into plasma cells. However, one important feature discriminates antibodies from all other immunoglobulin superfamily members: antibodies are the only family member that, in their monomeric form, are always at least bivalent if not multivalent (Johansen et al., 2000). Irrespective of the considerable structural diversity that can be found across the animal kingdom, even the antibodies discovered in the phylogenetically oldest living vertebrates, such as sharks and other cartilaginous fish, are bivalent. Intriguingly, camelids and sharks possess single-domain antibodies consisting of a heavy chain only, devoid of light chains (Könning et al., 2017), and truncated Fc-deficient versions are found in amphibians and cartilaginous fish, respectively, denominated immunoglobulin Y (IgY) and IgW (Rumfelt et al., 2004; Wang et al., 2012; Wei et al., 2009; Zhang et al., 2017). Irrespectively of this diversity, all of those antibodies have conserved their bivalent structure, highlighting the evolutionary significance of bivalency.

Typically only a small proportion of the antibodies elicited during infection can block virus entry into host cells in a process called neutralization. This activity of antibodies is commonly assessed in viral plaque reduction neutralization tests (PRNT) and herein is referred to as neutralizing. While neutralizing activity of monoclonal antibodies (mAbs) is usually a fair predictor of their protective efficacy (Corti and Lanzavecchia, 2013), neutralizing but non-protective antibody clones have also been identified (Cross et al., 2016; Oswald et al., 2007). Moreover, also non-neutralizing antibodies (nnAb) are often protective *in vivo*, which can involve Fc gamma receptor (FcγR)-dependent and -independent mechanisms (Abreu-Mota et al., 2018; Mayr et al., 2017a, 2017b; Richter and Oxenius, 2013; Straub et al., 2013). Antibody binding can activate the complement system by interaction with its component 1q (C1q) to inactivate cell-free virus or lyse infected cells (Bernet et al., 2003; Cooper and Nemerow, 1983). Antibodies bound to virus-infected cells can induce antibody-dependent cell-mediated cytotoxicity (ADCC) and antibody-dependent cellular phagocytosis (ADCP) through interactions with classical FcγRs on the surface of effector cells (Bournazos et al., 2015). Also, cellular interactions by means of non-classical Fc receptors, such as the neonatal Fc receptor (FcRn) (Junghans and Anderson, 1996; Montoyo et al., 2009), the cytoplasmic Fc receptor Tripartite motif containing 21 (TRIM21) (Caddy et al., 2021; Hauler et al., 2012; James et al., 2007; Mallery et al., 2010), and SIGN-R1 (binding only sialylated antibodies) (Anthony et al., 2008; Nimmerjahn and Ravetch, 2008), can contribute to antibody efficacy. Finally, cell culture studies on a broad range of viruses, such as influenza virus (Dowdle et al., 1974), herpes virus (Driscoll et al., 1977), vaccinia virus (Vanderplasschen et al., 1997), rubella virus (Cordoba et al., 2000), Marburg virus (MARV) (Kajihara et al., 2012), and Chikungunya virus (CHIKV) (Fox et al., 2015; Jin et al., 2015, 2018; Jin and Simmons, 2016), have documented that neutralizing as well as non-neutralizing envelope protein-specific antibodies can inhibit the release of viral particles from infected cells and/or tether virions to the cell surface from which they are released. The potential significance of this mechanism for antibody-mediated *in vivo* protection remains, however, elusive. Interestingly, monovalent Fab fragments often potently neutralize viruses in cell culture, whereas bivalent antibody binding was strictly required for inhibition of MARV and CHIKV release from infected cells (Jin et al., 2018; Kajihara et al., 2012). However, the significance of antibody bivalency for *in vivo* protection remains largely unexplored.

Lymphocytic choriomeningitis virus (LCMV) is an enveloped negative-stranded RNA virus that has long and widely been used as a versatile model agent to investigate virus-host interactions in mice (Zinkernagel, 2002). Irrespective of the undisputed key contribution of cytotoxic CD8+ T cell responses to LCMV control (Fung-Leung et al., 1991), specific antibodies produced with the aid of follicular T helper cells are essential for the resolution of chronic infection (Bergthaler et al., 2009; Greczmiel et al., 2017; Harker et al., 2011). Intriguingly, however, neutralizing antibodies (nAbs) are only formed several weeks after the infection is resolved. While these observations point to a key contribution of nnAbs in virus control (Bergthaler et al., 2009; Richter and Oxenius, 2013; Straub et al., 2013), the underlying mechanisms remain incompletely defined.

Here we report that bivalent antibody binding enables FcγR-independent protection by non-neutralizing LCMV glycoprotein (GP)-specific Abs and correlates with the antibodies' ability to tether virions to the infected cell surface. Our observations suggest bivalency-dependent inhibition of virion release as an archetypical and highly conserved antiviral effector mechanism, which may account for a substantial proportion of nnAb and even nAb efficacy.

## Results

### Neutralization, Fcγ receptors, and complement are dispensable for antiviral protection by a monoclonal antibody

**Figure 1:**
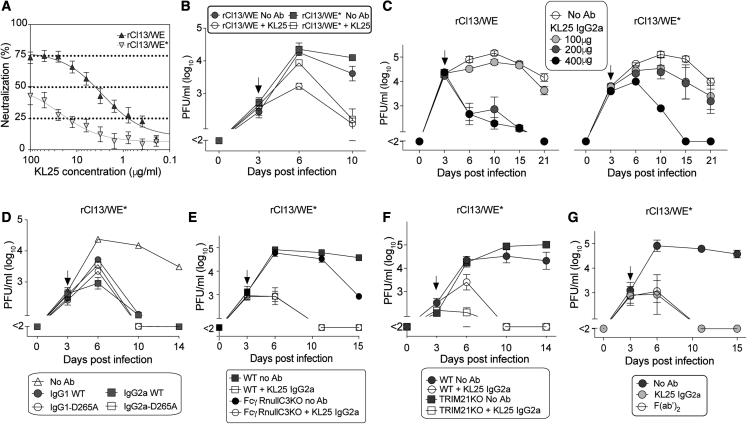
Passive antibody treatment suppresses viremia independently of antibody PRNT potency and Fc-mediated effector functions (A) PRNT activity of rKL25 against rCl13/WE and rCl13/WE^∗^. (B–G) We infected WT mice (B–D, G) and mice of the indicated genotypes (E and F) with rCl13/WE or rCl13/WE^∗^ intravenously (i.v.) and treated them 3 days later (arrow) with 300 μg of either antibody format. WT mice in (G) were treated either by a single dose of KL25 IgG2a or with F(ab')_2_ fragments in a repeated dosing regimen to mimic the washout of KL25 IgG2a (see Figure S1K). Controls were given no antibody (no Ab). Viremia was monitored over time. (B and C) Comparable efficacy of KL25 against rCl13/WE and rCl13/WE^∗^. (D) Efficacy of FcγR- and C1q-blind (D265A mutant) antibody independently of antibody isotype. (E and F) KL25 efficacy in FcγRnullC3KO and TRIM21KO mice. Symbols represent the mean ± SEM of three replicate samples (A), or four mice per group (B, C, F, and G), or three mice per group (D and E). One representative experiment of two is shown.

To examine antibody effector mechanisms in a chronic infection setting of mice, we used a recombinantly engineered LCMV strain, clone 13, which expresses the envelope glycoprotein of the WE strain (herein referred to as rCl13/WE; Penaloza-MacMaster et al., 2015; Sommerstein et al., 2015). This recombinant virus offers the advantage that chronic infection can be studied in conjunction with variants of the WE glycoprotein, for which virus-antibody interactions have been well characterized (Hangartner et al., 2006; Seiler et al., 1998b). rCl13/WE viremia lasts for ∼30 days (Figure S1A), and viral clearance depends on the ability of mice to mount virus-specific antibody responses (Sommerstein et al., 2015). nAbs are only produced between 40 and 80 days after infection, however, such that, at the time when viremia subsides, i.e., around day 30, the serum of mice is still devoid of detectable neutralizing capacity (Figure S1B) as determined by PRNT. To investigate mechanisms of LCMV nnAb protection *in vivo* (Bergthaler et al., 2009; Caddy et al., 2021; Richter and Oxenius, 2013; Straub et al., 2013), we engineered an rCl13/WE virus carrying an N121K point mutation in GP1 (rCl13/WE^∗^). rCl13/WE^∗^ was approximately 40–60-fold less sensitive to neutralization by the widely studied monoclonal antibody (mAb) KL25 than rCl13/WE (Figures 1A and S1D, (Hangartner et al., 2006)), and KL25 binding affinity was ∼8-fold reduced (Table S1). To test if differential PRNT activity correlated with antiviral efficacy of passive antibody therapy, we infected mice with either rCl13/WE or rCl13/WE^∗^ and treated them with 300 μg of KL25 3 days later. KL25 therapy suppressed both viruses *in vivo* with similar kinetics (Figure 1B). Isotype control antibody failed to suppress viremia, attesting to the specificity of the antiviral antibody effects observed (Figure S1C). To better assess potential quantitative differences in protective KL25 efficacy as a function of the viral GP, we titrated the KL25 antibody dose given to mice. Only approximately twice as much KL25 was required to clear rCl13/WE^∗^ infection as was sufficient to eliminate rCl13/WE (Figure 1C). Hence, an 8-fold higher binding affinity and 30–50-fold higher neutralizing potency against rCl13/WE translated into only ∼2-fold higher therapeutic *in vivo* potency, questioning neutralization as a main effector mechanism of anti-LCMV antibodies *in vivo*. Besides neutralization, FcγR-mediated mechanisms have been reported to contribute to antiviral antibody efficacy (Bournazos and Ravetch, 2017). To account for differential FcγR-binding properties, we recombinantly expressed the KL25 mAb in either IgG1 or IgG2a format (KL25-IgG1, KL25-IgG2a). Additionally, we introduced the D265A mutation into the CH2 domain of either heavy chain, which abrogates antibody Fc interactions with FcγRs and complement component 1q (C1q, Baudino et al., 2008; KL25-IgG1-D265A and KL25-IgG2a-D265A). Neither the isotype nor the D265A modification of KL25 affected the antibodies' binding to WE or WE^∗^ GP (Figure S1E), and, intriguingly, all four antibody formats afforded comparable protection independently of their subclasses and Fc receptor-binding ability (Figure 1D). In order to corroborate FcγR- and complement-independent antibody protection, we exploited FcγRnull/C3KO mice, which are deficient in all four classical Fcγ receptors (I, IIb, III, IV) as well as complement component 3 (C3). We found that, upon KL25 treatment, both wild-type (WT) and FcγRnull/C3KO mice cleared rCl13/WE^∗^ infection with similar kinetics (Figure 1E). C1q deficiency did not prevent KL25-mediated virus control either (Figure S1F). The cytoplasmic Fc receptor TRIM21 can degrade antibody-coated capsids of viruses after cell entry (McEwan and James, 2015) and has been reported to contribute to protective effects of LCMV nucleoprotein (NP)-specific antibodies (Caddy et al., 2021). Thus, we infected TRIM21-deficient animals with rCl13/WE^∗^ and treated them with rKL25-D265A, which was similarly effective as in WT animals and thus argued against a key role of TRIM21 in LCMV envelope glycoprotein-specific antibody protection (Figure 1F). SIGN-R1, a transmembrane C-type lectin, has been shown to function as an alternative Fc receptor (Kang et al., 2004). To test a potential contribution of SIGN-R1 effects to KL25 *in vivo* efficacy, we used a SIGN-R1 blocking antibody. Upon rCl13/WE^∗^ infection, SIGN-R1-blocked animals had slightly higher viremia than untreated controls, but KL25-D265A treatment cleared the infection in both groups with similar kinetics (Figure S1G). Recent reports have emphasized the “sweeping” activity of antibody Fc interactions with the neonatal Fc receptor (FcRn) to play an important role in antibody-mediated pathogen clearance from the blood stream (Roopenian and Akilesh, 2007). Hence, we engineered a KL25 antibody with mutations at the CH2/CH3 interphase (I253A, H435A, H436A, KL25-IHH), which are known to prevent FcRn (Kim et al., 1994) and TRIM21 (McEwan and James, 2015) binding. *In vivo* protection experiments with KL25-IHH were complicated by the short half-life of this antibody, which was predicted owing to its failure to bind and be salvaged by FcRn in the endosomal compartment (Roopenian and Akilesh, 2007). Hence, we established a repeated administration regimen for KL25-IHH, resulting in serum antibody concentrations over time that mimicked the washout of WT KL25 in rCl13/WE^∗^-infected mice (Figure S1H). When administered in this manner, KL25-IHH cleared rCl13/WE^∗^ infection comparably with WT KL25 (Figure S1I). Analogous experiments were conducted in FcRn-deficient mice (Figure S1J), altogether demonstrating that, when compensating for the shortened antibody half-life in the absence of FcRn binding (Figure S1H), the sweeping activity of this receptor did not essentially contribute to the efficacy of KL25 therapy. Taken together, these results questioned a clear-cut role of the various known Fc domain-mediated effector functions to anti-LCMV antibody protection, but extensive redundancy in the various Fc-dependent antibody effector mechanisms could not be formally ruled out. Thus, we treated rCl13/WE^∗^-infected animals with F(ab')_2_ fragments of KL25, which are devoid of the Fc portion. Analogously to KL25-IHH, the failure to bind FcRn shortens the *in vivo* half-life of F(ab')_2_ fragments. Hence, F(ab')_2_ fragments were administered to mice by the same repeated dosing regimen as established for KL25-IHH (Figure S1K). Although the resulting serum concentration of F(ab')_2_ fragments was somewhat lower than the one of unmodified KL25 control antibody (Figure S1K), treatment with F(ab')_2_ fragments was as protective as WT KL25 full-length antibody (Figure 1G). Altogether, these findings indicated that Fc-mediated mechanisms were dispensable for KL25-mediated clearance of chronic LCMV infection in mice.

### KL25 efficacy is not due to an indirect immunostimulatory effect but relies on the prevention of viral spread

**Figure 2:**
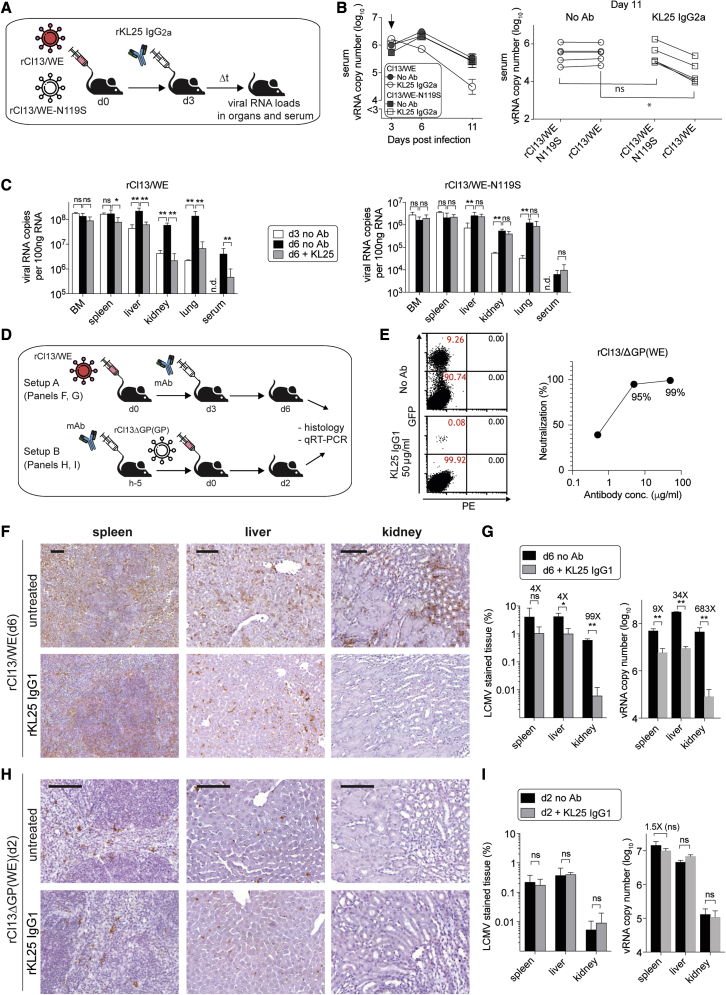
KL25 efficacy is not due to an indirect immunostimulatory effect but relies on the prevention of viral spread (A–D) (A) Schematic of the experiment in (B) and (C). We co-infected WT mice (n = 4) with a 1:1 mixture of rCl13/WE and rCl13/WE-N119S. KL25-IgG2a was administered 3 days later, serum was collected on d3, d6, and d11 (B), and organs were harvested on d3 and d6 (C). rCl13/WE and rCl13/WE-N119S RNA copy numbers in co-infected mice were individually quantified by RT-qPCR from serum (B) and organs (C). ^∗^p < 0.05, ^∗∗^p < 0.01 by unpaired Student's t test on log-converted values (B) and by one-way ANOVA with Dunnett's post-test comparing each group with the d6 + no Ab group (C). Arrow in (B) indicates Ab treatment. Symbols in (B, left) show the mean ± SEM of four mice per group, with individual d11 values shown in (B, right) and compared by Student's t tests. Bars in (C) represent the mean ± SEM of four mice. (D) Schematic of the experiments in (F) and (G) (setup A) and (H) and (I) (setup B). In setup A, mice were infected with rCl13/WE, KL25 was administered on d3, and organs were assessed on d6 (F, G). In setup B, KL25 was administered to mice, and replication-deficient rCl13ΔGP(WE) vector was injected i.v. 5 h later. Organs were analyzed on d2 (H, I). Control groups were without antibody treatment (no Ab). (E) FCNT verified that KL25 antibody neutralizes rCl13ΔGP(WE) vectors. Representative fluorescence-activated cell sorting (FACS) panels (left) and dose-dependent neutralization (right). (F and H) Virus-infected cells (LCMV NP-positive) in spleen, liver and kidney of rCl13/WE-infected (F) or rCl13ΔGP(WE) vector-inoculated (H) mice. (G and I) The percentage of LCMV antigen-stained tissue surface (left) and viral RNA copies by RT-qPCR (right). Antibody efficacy calculated as fold reduction of LCMV NP-positive tissue surface and viral RNA copy numbers, respectively, are indicated. Scale bars: 100 μm (F and H). Representative histology images from four individual mice are shown, two or three visual fields were analyzed per organ and animal. Bars in (G) and (I) represent the mean ± SEM of four mice per group. One representative experiment of two is shown for (B), (E), (G), and (I). Data in (C) are independently reproduced in Figure S2. ^∗^p < 0.05, ^∗∗^p < 0.01; statistical analyses were performed by unpaired Student's t tests on log-converted values.

Antibody coating of virions can augment other immune defense mechanisms, such as T cell responses (Bournazos et al., 2015; Caddy et al., 2021), which are important for the control of primary LCMV infection (Fung-Leung et al., 1991). Thus, we sought to test the possibility that KL25 treatment suppressed viral loads indirectly by potentiating other immune defense mechanisms rather than by direct antiviral effects. Along the lines of a previously established approach (Johnson et al., 2015), we set up a viral co-infection experiment, allowing us to differentiate direct antibody-mediated effects on virus loads from indirect immunostimulatory effects (Figure 2A). We inoculated mice with a mixture of rCl13/WE and a variant virus, which carries a point mutation that completely abrogates KL25 binding (rCl13/WE-N119S; Hangartner et al., 2006) (Table S1) but does not affect T cell recognition of the virus. A stretch of non-coding nucleotide differences (genetic tag) in the NP sequence of the two viruses allowed the individual enumeration of each virus' RNA copy numbers in the serum of co-infected animals by TaqMan RT-PCR (Johnson et al., 2015). We found that KL25 treatment selectively suppressed serum RNA levels of the KL25-sensitive rCl13/WE, whereas rCl13/WE-N119S, which competed against rCl13/WE in the same animal but was not bound by KL25, remained unaffected (Figure 2B). This virus-selective effect of KL25 indicated that its antiviral effect was predominantly a direct one, whereas indirect effects via other immune defense mechanisms, such as T cells, should have suppressed both viruses. Analogously to these results from blood, KL25 antibody treatment reduced rCl13/WE but not rCl13/WE-N119S levels in liver, kidney, and lung of mice on day 6 (Figure 2C). Interestingly, however, KL25 therapy exerted only modest or insignificant effects on viral RNA levels in bone marrow and spleen (Figure 2C). Similar results were obtained when mice were co-infected with rCl13/WE^∗^ and rCl13/WE-N119S, and/or when FcγR-blind KL25-IgG2a-D265A was used for therapy (Figures S2A and S2B). It is known that spleen and bone marrow are primary LCMV target organs, which are saturated with virus by day 3, while liver, kidney, and lung represent secondary target organs with viral loads still accumulating as chronic infection progresses (Richter and Oxenius, 2013). Accordingly, it appeared that KL25 was most effective at reducing viral loads in those organs, where viral loads increased between the day of treatment (day 3) and viral load measurement on day 6. KL25 effects in these viral co-infection experiments were quantitatively more modest than in the single infection setting studied in Figure 1, a difference that is presumably due to the diversion of the CD8 T cell response by the persisting rCl13/WE-N119S virus and/or the cells' resulting exhaustion (Zajac et al., 1998).

The differential effects of KL25 therapy on primary and secondary target organs led us to further examine and differentiate the impact of KL25 on virus entry into cells as opposed to antibody effects on virus spread, which also comprises potential antibody effects on virus release from infected cells (Cordoba et al., 2000; Dowdle et al., 1974; Driscoll et al., 1977; Jin et al., 2018; Jin and Simmons, 2016; Kajihara et al., 2012; Vanderplasschen et al., 1997). To study by histology the impact of antibody on viral spread, we infected mice with rCl13/WE on day 0, treated them with KL25 3 days later or left them untreated, followed by analysis on day 6 (Figure 2D, setup A). The number of virus-infected cells as determined by immunohistochemistry was significantly reduced in antibody-treated animals, both in liver and kidney (Figures 2F and 2G). TaqMan RT-PCR was performed to verify that viral load differences as assessed by immunohistochemistry were also evident in substantially lowered viral RNA copy numbers (Figure 2G). To selectively assess the effect of KL25 on virus entry, independently of potential antibody effects on virus release and consequent spread, we relied on rCl13ΔGP(WE) vectors. These single-round infectious particles infect cells by means of their membrane-incorporated WE envelope protein, which they acquire from producer cells during *in vitro* production. They do not, however, encode for GP and, hence, cannot form infectious progeny particles to spread *in vivo* (Bergthaler et al., 2010; Flatz et al., 2010). When assessed in a flow cytometry-based neutralization test using GFP-expressing rCl13ΔGP(WE) vectors as a test article (flow cytometry neutralization tests [FCNTs]; Figure S3A), up to 99% of vector particles were neutralized by KL25 in a concentration-dependent manner (Figure 2E). We administered KL25 prophylactically to mice and subsequently challenged them with rCL13ΔGP(WE). Tissues were analyzed 2 days after vector administration (Figure 2D, setup B) to determine viral antigen-positive cell density and vector RNA copies. These measurements were reflective of intracellular genome amplification as evident from control experiments with UV-inactivated vector particles (Figure S3B). Unlike in the setting of spreading infection (compare Figures 2F and 2G) and despite prophylactic KL25 administration resulting in serum concentrations in the range of 100 μg/mL (compare Figure S1H), there was no significant reduction in LCMV antigen-positive cell densities in either spleen, liver, or kidney, and also an RT-PCR-based assessment of viral RNA failed to demonstrate a clear impact of KL25 on vector entry *in vivo* (Figures 2H, 2I, and S3B). These observations showed a clear discrepancy between KL25 potently inhibiting vector entry *in vitro* but not *in vivo* (compare Figures 2E and 2H). While the above experiments were conducted using WE glycoprotein-pseudotyped rCl13ΔGP vectors (rCl13ΔGP(WE)), analogous results were obtained when rCl13ΔGP vectors were pseudotyped with the WE^∗^ glycoprotein for administration to mice (rCl13ΔGP(WE^∗^); Figure S3C). Altogether, these findings indicated that the therapeutic efficacy of KL25 in rCl13/WE and rCl13/WE^∗^ infection relied substantially on the inhibition of virus dissemination, whereas *in vivo* effects on viral cell entry appeared modest at best.

### KL25 inhibits the release of viral particles from cultured cells

**Figure 3:**
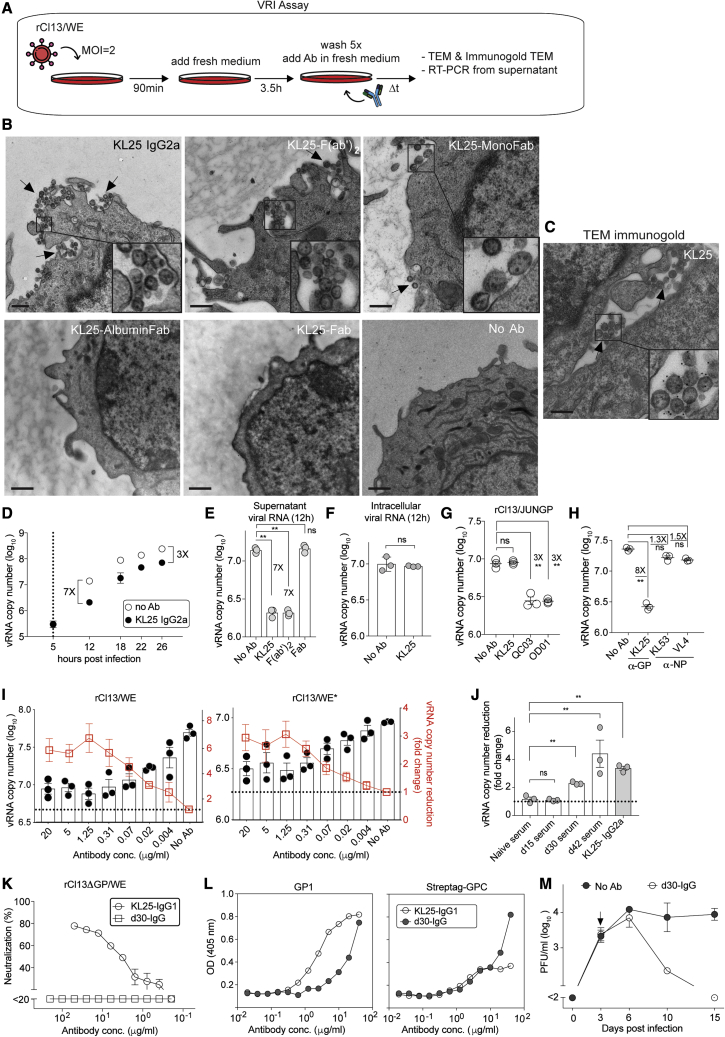
Bivalent but not monovalent antibody molecules tether virions to the infected cell surface and inhibit virion release (A) Experimental design of the VRI assay. (B–D) KL25 antibody formats were tested in a VRI assay on RAW264.7 cells. Twelve hours post infection the cells were processed for TEM (B) or immunogold TEM (C) to assess cell-surface-tethered virions. Tethered virions as shown in representative images were found in about three to five cells per 100 KL25-, F(ab')_2_- or MonoFab-treated cells. This variability is presumably due to compartmentalized virion release and imperfect coverage of the cell surface compartments by the ultrathin TEM sections. Conversely, not a single tethered virion was found in >200 cells of untreated or Fab-treated samples. (D) Viral RNA copies in cell culture supernatant were monitored. (E) Bivalent KL25-IgG2a and F(ab')_2_ but not Fab molecules are active in VRI. (F) Intracellular viral RNA copies in a VRI assay. (G) Junin virus GP-specific mAbs QC03 and OD01 but not LCMV-GP-specific KL25 inhibit the release of Junin-GP-expressing LCMV (rCl13/JUNGP). (H) The GP-specific mAb KL25 but not the NP-specific mAbs KL53 and VL4 are active in VRI assays. (I) VRI assay on rCl13/WE- or rCl13/WE^∗^-infected RAW264.7 cells document similar KL25 dose response. (J) VRI activity of polyclonal sera collected at the indicated time points after rCl13/WE infection and pre-diluted 1:50. Each experiment was performed with three technical replicates. VRI activity calculated as fold-change compared with no Ab (D–I) or naive serum (J). (K and L) d30-IgG (K-M) in FCNT (K) and in ELISA (L) against GP1 and GP-C of WE. (M) Mice were infected with rCl13/WE, treated with d30-IgG on d3 (arrow), or were left untreated (no Ab) and viremia was monitored. (B–D) One representative image out of several regions analyzed in at least three TEM images captured. Symbols show the mean ± SEM of three technical replicates in (D), (K), and (I) (red squares) or individual values in (E)–(J) and (L). Bars represent the mean ± SEM of three technical replicates in each group. The mean ± SEM of two mice is shown in (M). One representative experiment of two similar ones (F–M) or one out of three experiments (D and E) is shown. Scale bars in (B) and (C): 500 nm. ^∗^p < 0.05, ^∗∗^p < 0.01 by one-way ANOVA of log-converted RNA copy numbers.

These *in vivo* findings prompted us to study a potential impact of KL25 on the release of virions from infected cells in culture. LCMV in mice targets predominantly myelomonocytic cells (Homann et al., 2004). Hence, we infected the macrophage cell line RAW264.7 with rCl13/WE at high multiplicity of infection (MOI = 2), washed away residual inoculum and then overlaid the culture with KL25-containing medium to determine a potential inhibitory effect of antibody on virion release (virion release inhibition [VRI] assay; Figure 3A). Transmission electron microscopy (TEM) was conducted 12 h after infection, when new viral infectivity becomes detectable in supernatant, revealing that KL25 tethered newly produced virions to the cell surface (Figure 3B). Immunogold staining confirmed that the membrane-tethered virions on the cell surface were indeed antibody bound (Figure 3C). To quantitatively assess VRI in our VRI assay (Figure 3A), viral genome copies in the supernatant of infected cells were quantitated by TaqMan RT-PCR. The addition of KL25 to the culture medium reduced virion RNA in supernatant by approximately 7-fold at 12 h after infection, an effect that was still noticeable at 26 h, albeit somewhat less prominently (3-fold; Figure 3D). An analogous tethering of virions to the infected cell surface and comparable VRI activity were noted when KL25 F(ab')_2_ fragments were used (Figures 3B and 3E). In contrast, monovalent Fab fragments and isotype control antibody did not significantly reduce virion release, and cell surface-tethered virions could not be found (Figures 3B, 3E, and S4A). These findings were in line with published reports on MARV and CHIKV, demonstrating that a bivalent antibody format but not monomeric Fab structures tethered viral particles to the infected cell surface (Kajihara et al., 2012) and effectively prevented virion release (Fox et al., 2015). To validate the VRI assay format, we verified that cells infected with a genetically tagged rCl13/WE-N119S virus (not recognized by KL25) could not be superinfected by rCl13/WE (Figure S4B). This was in line with published reports about LCMV superinfection exclusion (Ellenberg et al., 2007) and ruled out the possibility that supposed KL25 effects on virion release reflected antibody interference with viral spread in the culture. Moreover, RNAse resistance tests conducted prior to and after detergent treatment verified that the vast majority of viral RNA detected in culture supernatants of VRI assays was lipid enveloped and thus corresponded to virions rather than infected cell debris (Figure S4C). Importantly also, the intracellular accumulation of LCMV RNA was unaltered by the addition of KL25 to the culture medium, indicating that the release of virions but not intracellular viral genome amplification was affected (Figure 3F). VRI assays were also conducted in the peritoneal macrophage-derived cell line IC-21, with analogous results to RAW264.7 cells (Figure S4D). In line with the unimpaired activity of F(ab')_2_ fragments, the VRI effects of KL25-IgG1, KL25-IgG2a, and KL25-IgG2a-D265A were indistinguishable (Figure S4E). VRI activity was also noted for two Junin virus glycoprotein (JUNV-GP)-specific mAbs (Figure 3G), generalizing the concept of VRI as an effector mechanism of arenavirus-nAbs. LCMV (NP-binding antibodies can afford passive protection against LCMV *in vivo* (Richter and Oxenius, 2013; Straub et al., 2013) but did not significantly affect virion release (Figure 3H), suggesting that VRI activity was linked to antibody envelope binding. VRI tests were also conducted using serial dilutions of KL25 on either rCl13/WE- or rCl13/WE^∗^-infected cells and showed comparable 50% inhibitory concentrations (IC_50_; Figure 3I). These results contrasted with ∼50-fold higher PRNT IC_50_ concentrations of KL25 against rCl13/WE^∗^ than against rCl13/WE (see Figures 1A and S1D) but matched comparable dose-dependent antiviral protection in mice (see Figures 1B and 1C). Of special note, the VRI IC_50_ concentration of KL25 was in the range of 0.1μg/mL for both viruses, thus substantially lower than the PRNT IC_50_ of ∼2μg/mL and ∼100 μg/mL against rCl13/WE and rCl13/WE^∗^, respectively (Figure 3I, compare Figure 1A). Still, the VRI effect of KL25 against rCl13/WE^∗^ plateaued at somewhat lower levels than for rCl13/WE, which might be related to the lower-affinity interaction (Figure 3I). To quantitatively better visualize these VRI effects, Figure 3I and subsequent figures report VRI assay data as viral RNA fold change to untreated control wells. The above findings raised the possibility that VRI assays offered a particularly sensitive method for the detection of protective, viral-envelope-specific antibody activity in LCMV-convalescent sera. Indeed, we found that mouse sera collected as early as 30 days after rCl13/WE infection exhibited significant VRI activity, while PRNT activity was undetectable (Figure 3J, compare Figure S1B). To better characterize this early VRI response, we purified polyclonal IgG from mice on day 30 after rCl13/WE infection (d30-IgG). Although inactive in FCNT and PRNT (Figures 3K and S5), d30-IgG bound not only the recombinantly expressed full-length extracellular domain of WE glycoprotein but also the receptor-binding LCMV-GP1 domain when expressed as a subunit (Figure 3L). Moreover, d30-IgG potently suppressed viral replication when passively administered to mice (Figure 3M). These observations suggested VRI as a mechanistic correlate of protection of non-neutralizing d30-IgG.

### KL25-MonoFab exhibits Fcγ-dependent VRI activity and suppresses viremia in an FcγR-dependent manner

**Figure 4:**
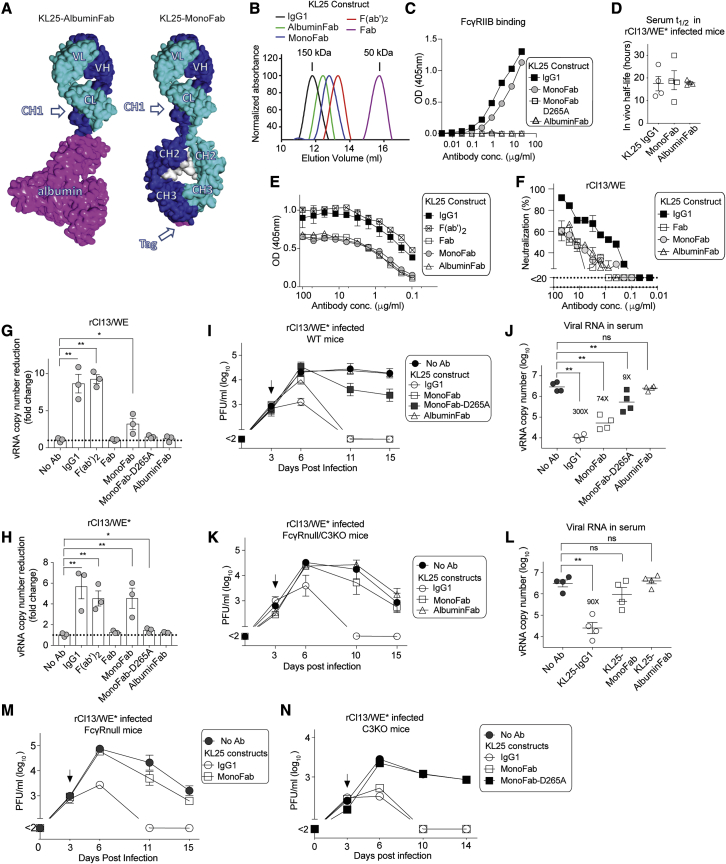
KL25-MonoFab exhibits Fcγ-dependent VRI activity and suppresses viremia in an FcγR-dependent manner (A) Schematic of engineered monovalent antibodies. In AlbuminFab, albumin substitutes for the heavy chain CH2-CH3 domains, with a flexible linker to the VH-CH1 domain. MonoFab is a heterodimer of a modified heavy chain and a light chain derivative, in which the mouse IgG1 CH2 and CH3 domains are fused to the kappa light chain, connected by a part of the hinge domain (Figure S6). (B) KL25 MonoFab and AlbuminFab were purified by affinity chromatography followed by size exclusion chromatography and were reanalyzed by size exclusion chromatography (Figure S6D). A superposition of their elution profiles with elution maxima of KL25 IgG1, Fab2, and Fab molecules is displayed for a comparison of relative molecular weights. (C) Binding of the indicated KL25 constructs to mouse FcγRIIb in ELISA. Symbols indicate the mean ± SD of two independent measurements. (D) WT mice (n = 4) were treated with 300 μg of rKL25, MonoFab, or AlbuminFab 3 days after rCl13/WE^∗^ infection. Serum concentrations were determined 24 and 48 h later to calculate the molecules' *in vivo* half-life under infection conditions. (E) GP1 binding was assessed by ELISA. (F) rCl13/WE PRNT activity of the indicated KL25 constructs. (G and H) We performed VRI assays using rCl13/WE (G) or rCl13/WE^∗^ (H) and the indicated antibody constructs. (I–N) WT (I and J) and FcγRnull/C3KO (K and L) mice were infected with rCl13/WE^∗^ on d0 and were treated with KL25 IgG1 (300 μg), MonoFab (300 μg), MonoFab-D265A (500 μg), or AlbuminFab (500 μg) on d3 (arrow). MonoFab-D265A and AlbuminFab were dosed higher than KL25 and MonoFab to exclude the possibility that a lack of efficacy was due to borderline dosing. Viremia was determined (I and K). Viral RNA copy numbers in serum were quantified at d10 and d11, respectively (J and L). The fold reduction in viral RNA load compared with untreated animals is indicated. Representative results from two independent experiments are shown. Symbols in (E) and (F) indicate the mean ± SEM of three independent measurements; symbols in (G) and (H) show individual cell culture wells and symbols in (D), (J), and (L) represent individual mice. Bars in (G) and (H) show the mean ± SEM of three technical replicates. Symbols in (I), (K), (M), and (N) represent the mean ± SEM of four mice per group. ^∗^p < 0.05, ^∗∗^p < 0.01 compared with no antibody controls (no Ab), as determined by one-way ANOVA with Dunnett's post test, conducted on log-converted values.

Aiming to assess whether VRI contributes to antibody-mediated protection *in vivo*, we considered that antibody bivalency seems key for this antibody effector mechanism (compare Figures 3B and 3E) (Fox et al., 2015; Kajihara et al., 2012). The *in vivo* half-life of Fab fragments and related monovalent antibody formats ranges, however, in the order of a few minutes only (Kalinke et al., 1996), precluding their use for experiments aimed at testing the relationship between antibody bivalency and protective efficacy in mice. Hence, we engineered a monovalent but full-length Fc-bearing KL25 antibody by attaching CH2 and CH3 domain to its kappa light chain (MonoFab; Figures 4A, S6A, and S6C) and by introducing amino acid exchanges in the CH3 domain interface of the antibody Fc region promoting heterodimer formation by the engineered proteins. The amino acid exchanges result in altered charge polarity across the Fc dimer interface such that co-expression of electrostatically matched Fc chains favors the desired Fc heterodimer formation during production (see STAR Methods; Gunasekaran et al., 2010) (Figure S6C). In a second independent construct (AlbuminFab), the CH2 and CH3 domain of the KL25 heavy chain were exchanged for albumin, connected by a flexible linker (Figures 4A and S6B). MonoFab and AlbuminFab were purified by affinity chromatography followed by preparative size exclusion chromatography. Subsequently they were reanalyzed to confirm they were monomeric, exhibited the expected ∼100 kDa and ∼120 kDa molecular weight, respectively (Figures 4B and S6D), and contained their respective two amino acid chains at approximately equimolar ratio (Figures S6E and S6F). MonoFab bound to FcγR IIb and III, the two murine FcγRs bound by mouse IgG1 (Dekkers et al., 2017). Compared with its parental IgG1 molecule, MonoFab binding to FcγRs was modestly reduced, supposedly owing to the CH3 mutations introduced (Shields et al., 2001). In contrast and as expected, D265A-mutant MonoFab and AlbuminFab failed to detectably bind either FcγR (Figures 4C and S6G). Analogously to the Fc portion of IgG molecules, albumin binds to FcRn and thereby is salvaged from endosomes. Accordingly and as expected, the half-life of KL25-MonoFab and KL25-AlbuminFab in rCl13/WE^∗^-infected mice was indistinguishable from KL25-IgG1 (Figure 4D), rendering these constructs suitable to assess the impact of antibody bivalency on antiviral protection *in vivo*. Unlike the MonoFab construct, however, AlbuminFab molecules are devoid of an Fc portion, thus precluding interactions with classical Fcγ receptors. By means of their Fab domain, KL25-MonoFab and KL25-AlbuminFab binding to recombinant LCMV-GP1 in ELISA resembled the one of WT KL25 antibody (Figure 4E). As expected based on their differential stoichiometry, however, the monovalent antibody constructs yielded only approximately 50% of the total optical density (OD) signal of WT KL25 antibody when a light-chain-specific antibody was used for detection (Figure 4E). Conversely, MonoFab and AlbuminFab exhibited ∼30-fold reduced neutralizing activity in PRNT assays (Figure 4F). These *in vitro* binding and neutralization characteristics of MonoFab and AlbuminFab molecules recapitulated the behavior of KL25 Fab fragments.

Next, we tested the monovalent KL25-MonoFab and KL25-AlbuminFab constructs in VRI assays using either rCl13/WE or rCl13/WE^∗^. To our surprise, the monovalent KL25-MonoFab construct exhibited significant VRI activity (Figures 4G and 4H). In line with this observation, tethered virions were found by TEM on the surface of KL25-MonoFab-treated cells, albeit in somewhat smaller aggregates than under KL25-IgG2a treatment conditions (Figure 3B). On the contrary, KL25-AlbuminFab exerted no detectable inhibitory effect on virion release (Figures 4G and 4H), and cell surface-tethered virions were not detected by TEM (Figure 3B). This differential behavior of the MonoFab and AlbuminFab formats was intriguing given they were both monovalent. We speculated that MonoFab, by means of its intact Fc portion, might engage Fcγ receptors on the cell surface to compensate for its monovalent binding to the viral GP (Figure S6H). Hence, we generated a D265A variant of KL25-MonoFab, to disable the molecule's Fcγ receptor binding. In keeping with the above hypothesis, the D265A mutation largely abrogated the VRI activity of the KL25-MonoFab construct (KL25-MonoFab-D265A; Figures 4G and 4H).

To assess whether the *in vivo* efficacy of antibodies depends on bivalency and/or Fcγ receptor interactions, we infected WT mice with rCl13/WE^∗^ and treated them with either one of our monovalent antibody formats 3 days later. When mice were given KL25-MonoFab, viral infectivity in blood became undetectable by day 11, analogously to animals treated with bivalent WT KL25 antibody (Figure 4I). In contrast, viremia persisted and was indistinguishable from untreated controls when KL25-AlbuminFab was administered to mice. Viremia persisted also in KL25-MonoFab-D265A-treated animals, albeit at somewhat reduced levels. These observations were independently confirmed by TaqMan RT-PCR measurements of viral RNA copies in serum (Figure 4J). Regular KL25 IgG suppressed viral RNA levels ∼300-fold by day 11 and KL25-MonoFab treatment was only ∼4-fold less potent (74-fold reduction). In contrast, KL25-AlbuminFab did not measurably reduce viral RNA copies, and KL25-MonoFab-D265A afforded a 9-fold reduction only. This hierarchy of antiviral potency was largely reflective of the hierarchy observed in VRI assays (compare Figures 4G and 4H). We corroborated these findings by testing the ability of KL25-MonoFab to clear rCl13/WE^∗^ infection in FcγRnull/C3KO animals. Indeed, unlike in WT mice, where MonoFab treatment promptly cleared viremia, it failed to do so in FcγRnull/C3KO mice (Figure 4K). Also, when assessed by TaqMan RT-PCR, we failed to detect a consistent reduction of viral RNA copies in the blood of MonoFab-treated FcγRnull/C3KO animals (Figure 4L). This contrasted with the 74-fold reduction upon MonoFab treatment of WT mice (compare Figure 4J). To determine the individual contribution of Fcγ receptors and/or complement to the antiviral efficacy of KL25-MonoFab, we tested its efficacy in mice lacking either all four classical FcγR receptors (FcγRnull mice) or C3 (C3KO mice). While KL25-MonoFab suppressed rCl13/WE^∗^ viremia in C3KO mice, it was ineffective in FcγRnull animals (Figures 4M and 4N). Taken together, these results suggested monovalent KL25-MonoFab protection *in vivo* relied largely on functional FcγR interactions, whereas bivalent antibody suppressed viral loads independently of its Fc portion, a pattern that correlated with these antibody formats' ability to inhibit virion release in cell culture.

### Additional LCMV-nAb clones corroborate that VRI activity correlates with *in vivo* protection

**Figure 5:**
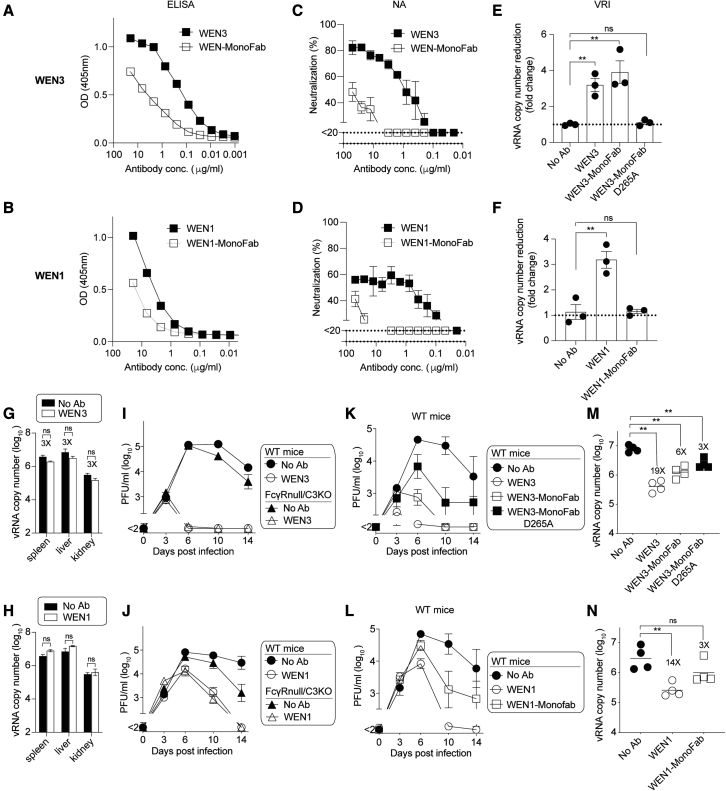
Additional LCMV-nAb clones corroborate that bivalency-dependent VRI activity correlates with *in vivo* protection (A–F) GP1 binding of the LCMV-nAbs WEN3 (A, C, and E) and WEN1 (B, D, and F) in IgG1 or MonoFab format (A and B), their PRNT potency (C and D), and VRI activity (E and F). Symbols in (A)–(D) show the mean ± SEM of three technical replicates, (E) and (F) show individual replicates with bars indicating the mean ± SEM. (G and H) WT mice were given 300 μg of WEN3 (G) or WEN1 (H), controls were without antibody treatment (no Ab, same control group reported in G and H). Five hours later, the animals were challenged with rCl13ΔGP(WE), and 2 days after vector administration we determined viral RNA copies in tissues. Antibody efficacy was calculated as viral RNA fold reduction compared with no Ab. (I–L) FcγRnull/C3KO and WT mice were infected with rCl13/WE on d0 and were treated with the indicated antibody constructs (300 μg) on d3 or left untreated (no Ab). (M and N) Viremia was monitored. Viral RNA copy numbers in serum on d6. Fold reduction compared with no-Ab controls is indicated. Representative results from two independent experiments are shown. Symbols and bars in (G)–(L) represent the mean ± SEM of four mice, symbols in (M) and (N) show individual animals. ^∗^p < 0.05, ^∗∗^p < 0.01 as determined by Student's t tests (G and H) and by one-way ANOVA followed by Dunnett's post test (E, F, M, and N), conducted on log-converted values.

To test the general validity of the observations made with the KL25 mAb, we converted two additional LCMV-nAbs (WEN3, WEN1) into the MonoFab format. WEN3-MonoFab and WEN1-MonoFab exhibited LCMV-GP1 binding curves largely parallel to those of their parental bivalent antibodies but reached lower OD values, a pattern reminiscent of the KL25-MonoFab binding behavior (Figures 5A and 5B; compare Figure 4E). In further analogy to the monovalent KL25 antibody formats, WEN3-MonoFab and WEN1-MonoFab exhibited substantially lower PRNT potency than their respective WT antibody counterparts (Figures 5C and 5D; compare Figure 4F). In terms of VRI activity, the potency of WEN3-MonoFab was comparable with its parental bivalent antibody. Analogously to KL25-MonoFab, this activity of WEN3-MonoFab was abrogated when FcγR interactions were disabled by the D265A mutation (WEN3-MonoFab-D265A; Figure 5E). In remarkable contrast to WEN3-MonoFab and KL25-MonoFab, however, WEN1-MonoFab failed to measurably inhibit virion release while the bivalent WEN1 antibody exerted significant VRI activity. When given prophylactically to mice, followed by rCl13ΔGP(WE) single-round vector administration (see Figure 2D, setup B, for experimental layout), neither WEN3 nor WEN1 significantly reduced vector RNA loads in spleen, liver, or kidney (Figures 5G and 5H). This indicated that, analogously to KL25 (compare Figures 2H and 2I), WEN3 and WEN1 effects on viral cell entry *in vivo* were modest at best. In contrast and in keeping with the VRI activity of these bivalent antibodies, WEN3 as well as WEN1 potently suppressed viremia in FcγRnull/C3KO mice, comparably with their effect in WT mice (Figures 5I and 5J). When tested therapeutically in rCl13/WE-infected WT mice, WEN3-MonoFab also promptly suppressed viremia, whereas WEN3-MonoFab-D265A therapy was of only intermediate efficacy (Figure 5K). TaqMan RT-qPCR measurements of viral RNA in serum confirmed this relative hierarchy of potency (Figure 5M). Therapeutic WEN1 administration almost completely suppressed infectious viral loads by day 10, whereas intermediate levels of viremia persisted in WEN1-MonoFab-treated mice (Figure 5L). Accordingly, viral RNA loads in the serum of WEN1-MonoFab-treated animals were not significantly lower than in untreated controls (Figure 5N). Taken together, these experiments revealed that certain antibodies, exemplified by WEN1, are virtually entirely dependent on bivalency for VRI activity and *in vivo* protection. Other antibodies, such as KL25 and WEN3, protect even in a monovalent format, provided they can establish functional FcγR interactions. In either case, *in vivo* protection correlated better with VRI activity than with PRNT potency in cell culture and, in the absence of FcγR interactions, protection depended largely on antibody bivalency.

## Discussion

Antibody multivalency is evolutionarily more conserved than Fc domains or variable light chains (Flajnik, 2018; Rumfelt et al., 2004; Wang et al., 2012; Wei et al., 2009; Zhang et al., 2017), raising the question of the underlying selection pressure and resulting evolutionary benefit. While the avidity-enhancing effect of multivalency is commonly acknowledged, our observations in the viral infection context indicate that multivalency can be dispensable for protection provided Fc-mediated effector functions are intact. In return, bivalent antibodies protected independently of FcγR-mediated effector functions, correlating with these antibodies' VRI activity. The conservation of antibody multivalency in all jawed vertebrates may, therefore, indicate that inhibition of virion release represents one of the evolutionarily most ancient antibody defense mechanisms.

Multivalent antigen binding can augment the avidity and thereby may potentiate neutralizing capacity (Hewat et al., 1998; Wang and Yang, 2010), but the steric arrangement of epitopes on the mature virion surface often precludes multivalent antibody binding (Dimmock and Hardy, 2004; Hewat et al., 1998; Klasse and Sattentau, 2002; Kwong et al., 2000) or requires hinge region mutations to form unusual antibody structures (Calarese et al., 2003; Williams et al., 2021). Such structural constrains on the virion also explain, at least in part, the superior protective efficacy of engineered bispecific anti-HIV antibodies with the ability to cross-link two different epitopes on the same protein (Wang and Yang, 2010). During virion maturation and budding from the cell surface, viral envelope proteins assume a less strictly ordered arrangement than on the mature cell-free virion, supposedly rendering them more vulnerable to antibody binding. The viral budding process may thus represent a window of opportunity for antibody-mediated envelope protein crosslinking, offering a potential explanation for the effectiveness of early LCMV-immune sera (d30-IgG) when tested in VRI but not in PRNT assays. In line with these considerations, the ∼8-fold differential equilibrium dissociation constant (KD) of KL25 binding to WE as compared with WE^∗^ translated into 30–50-fold differential PRNT activity but only ∼2-fold higher dose-dependent *in vivo* potency and similarly minor differences in VRI. This differential impact of antibody KD on different biological effects and assay readouts is likely explained by the bivalent IgG format used. Bivalent interactions have been shown to compensate in a readout-dependent manner for antibody off-rate differences such as those accountable for differential KL25 binding to WE and WE^∗^ (Table S1) (Wu et al., 2005).

Members of numerous virus families have developed envelope proteins with structural features such as glycan shields, which prevent or impair potent antibody neutralization of mature virions (Fafi-Kremer et al., 2010; Francica et al., 2010; Sommerstein et al., 2015; Wei et al., 2003). Our data suggest that nnAbs can inhibit viral release with similar efficiency to nAbs, and VRI activity correlated better with the antibodies' *in vivo* protective capacity against LCMV than did their neutralizing activity. The VRI activity of antibodies may thus represent an attractive goal for vaccination against HIV, hepatitis C virus, and other enveloped viruses, for which nAbs are difficult to induce for said structural reasons. Although likely non-sterilizing by nature, blunted viral dissemination owing to inhibition of virion release can allow other immune defense mechanisms to gain the upper hand (Bergthaler et al., 2009). Even LCMV-nAbs commonly fail to confer sterilizing immunity (Seiler et al., 1998b) (Figures 2H, 2I, 5G, and 5H).

Neutralizing activity of antibodies and of derived Fab molecules represents an excellent correlate of protection in a variety of viral diseases (Plotkin, 2010). According to our data and those of others (Dowdle et al., 1974; Driscoll et al., 1977; Fox et al., 2015; Jin et al., 2015; Kajihara et al., 2012; Klasse, 2014; Shariff et al., 1991), most neutralizing mAbs and polyclonal sera will also show activity in VRI assays. The long-standing positive correlation of virus neutralization and *in vivo* protection does not, therefore, contradict our findings and interpretations in any way (Cohen and Corey, 2017; Corti et al., 2017). In contrast and as exemplified by the d30 LCMV-immune IgG as well as by KL25 in the context of rCl13/WE^∗^ infection, VRI activity does not predict PRNT potency but often correlates with antiviral efficacy. For LCMV, we and others have shown that specific antibody responses are pivotal to control protracted infection, although nAbs often are detectable only weeks after the infection is resolved (Bergthaler et al., 2009; Sommerstein et al., 2015). While nnAb effects in influenza, cytomegalovirus (CMV), Ebola, and HIV-1 infections (Bournazos and Ravetch, 2017; Holl et al., 2009; Nimmerjahn et al., 2015) have been attributed to Fc-mediated effector functions (Horwitz et al., 2017; Mayr et al., 2017b; Nimmerjahn et al., 2015), nnAb anti-LCMV protection was consistently found to operate independently of FcγR or complement (Bergthaler et al., 2009; Richter and Oxenius, 2013; Straub et al., 2013). Compromised FcγR functioning owing to hypergammaglobulinemia and circulating immune complexes in the chronic phase of LCMV infection may at least partially account for these specific findings in the LCMV infection model (Hunziker et al., 2003; Wieland et al., 2015; Yamada et al., 2015). VRI may thus be particularly important under conditions where neutralization and FcγRs are inefficient, positioning antibody multivalency-dependent VRI activity as an evolutionarily ancient fail-safe mechanism.

### Limitations of the study

Most of the present work characterizes the mechanism of action of only three monoclonal antibodies, such that the conclusions may not be generally applicable. While KL25, WEN1, and WEN3 count among the most potent LCMV-nAbs identified to date (Eschli et al., 2007; Seiler et al., 1998a), only select readouts were reproduced with polyclonal infection-immune antibody preparations (d30-IgG). Accordingly it remains unknown whether this selection of mAbs is a fair representation of the diversity in the host's nAb response, both in terms of binding affinities and epitopes targeted.

We acknowledge further that only select experiments in our study included an antibody isotype control group, which can be preferable to untreated controls.

## STAR★Methods

### Key resources table

**Table:** 

REAGENT or RESOURCE	SOURCE	IDENTIFIER
**Antibodies**		

Goat anti-mouse IgG-HRP	Jackson Immunoresearch	Cat#111-035-062 RRID: AB_2338513
Goat anti-human IgG Fcgamma-specific	Jackson Immunoresearch	Cat#109-005-098 RRID: AB_2337541
Goat anti-mouse IgG (Fcgamma-specific) F(ab')_2_ -HRP	Jackson Immunoresearch	Cat#115-036-071 RRID: AB_2338525
Anti-mouse CD209b (clone 22D1)	BioXCell	Cat#BE0220
Biotinylated Goat Anti-Rat IgG	Vector Laboratories	Cat#BA-9401; RRID: AB_2336208
Goat anti-Rat IgG-HRP	Jackson Immunoresearch	Cat#112-035-003; RRID: AB_2338128
VL4 Rat anti-LCMV-NP	Dr. D.D. Pinschwer (Battegay et al., 1991)	N/A
KL53	Dr. D. D. Pinschewer (Zeller et al., 1988)	N/A
KL25-IgG1 (hybridoma derived)	Dr. D.D. Pinschewer (Bruns et al., 1983)	N/A
WEN1-IgG2a (hybridoma derived)	Dr. D.D. Pinschewer (Eschli et al., 2007)	N/A
WEN3-IgG2a (hybridoma derived)	Dr. D.D. Pinschewer (Seiler et al., 1998a; 1998b)	N/A
rKL25-IgG1	Dr. D. D. Pinschewer (Fallet et al., 2020)	N/A
rKL25-IgG2a	Dr. D. D. Pinschewer (Fallet et al., 2020)	N/A
rKL25-D265A	This paper	N/A
rKL25-IHH	This paper	N/A
rWEN1-IgG1	Dr. D. D. Pinschewer (Fallet et al., 2020)	N/A
rWEN3-IgG1	Dr. D. D. Pinschewer (Fallet et al., 2020)	N/A
KL25-MonoFab	This paper	N/A
KL25-MonoFab-D265A	This paper	N/A
KL25-AlbuminFab	This paper	N/A
WEN1-MonoFab	This paper	N/A
WEN3-MonoFab	This paper	N/A
WEN3-MonoFab-D265A	This paper	N/A
Monoclonal Anti-Junin Virus (clone QC03-BF11)	BEI Resources	Cat#NR-2566
Monoclonal Anti-Junin Virus (clone OD01-AA09)	BEI Resources	Cat#NR-2567
MOPC-21	BioXcell	Cat#BE0083; RRID: AB_1107784
37.7H mIgG1	This paper	N/A

**Bacterial and virus strains**		

LCMV rCl13/WE (recombinant virus)	(Sommerstein et al., 2015)	N/A
LCMV rCl13/WE^∗^ (recombinant virus)	This paper	N/A
LCMV rCl13ΔGP(WE) (viral vector)	(Flatz et al., 2010)	N/A
LCMV rCl13ΔGP(WE^∗^) (viral vector)	This paper	N/A
LCMV rCl13/JUNGP (recombinant chimeric virus)	(Sommerstein et al., 2015)	N/A

**Chemicals, peptides, and recombinant proteins**		

Biotinylated mFcγRIIB/CD32	Sino Biological	Cat#50030-M27H-B
Biotinylated mFcγRIII/CD16	Acro Biosystems	Cat#FC6-M82E0
Strep-Tactin	IBA	N/A
ABTS Pierce	Thermo	Cat#34026
DAB	DAKO	Cat#K5001
DAPI	Invitrogen	Cat#D3571
Streptavidin	IBA Lifesciences	Cat#12853186
Streptavidin-Peroxidase	DAKO	Cat#P0397
Glutaraldehyde	Sigma	Cat#G5882
Paraformaldehyde	Fisher	Cat#30525-89-4
Sodium cacodylate	Sigma	Cat#C4945
Osmium Tetroxide	Electron Microscopy Sciences	Cat#22400
UranyLess Uranyl Acetate	Electron Microscopy Sciences	Cat#22409
Acetone	Electron Microscopy Sciences	Cat#15056
Epon812 resin	Electron Microscopy Sciences	Cat#14120
BSAc	Aurion Immunogold	Cat#900.099
10nm gold particles	BBInternational	Cat#EM. GMHL10
Mayer’s hemalum solution	Merck	Cat#109249
TRIzol Reagent	Thermo Fisher	Cat#15596018
Qiazol	Qiagen	Cat#79306
Pepsin	Sigma	Cat#P6887
WE-GP-StreptagII	(Sommerstein et al., 2015)	N/A
WE^∗^-GP-StreptagII	This paper	
WE-GP1-Fc	(Eschli et al., 2007)	N/A

**Critical commercial assays**		

QIAamp viral RNA mini kit	Qiagen	Cat#52906
RNeasy 96 Universal Tissue Kit	Qiagen	Cat#74881
SuperScript III Platinum One-Step qRT-PCR Kit	Invitrogen	Cat#11732088
IgG Fab preparation kit	Pierce	Cat#44980

**Deposited data**		

Raw and Analyzed Data	This Paper	Zenodo: https://doi.org/10.5281/zenodo.5750060

**Experimental models: Cell lines**		

Mouse: MC57G cells	ATCC	Cat#CRL-2295 RRID: CVCL_4985
Hamster: BHK-21 (Clone 13)	ECACC	Cat#85011433 RRID: CVCL_1915
Mouse: NIH/3T3	ATCC	Cat#CRL-1658 RRID: CVCL_0594
Human: 293T	ATCC	Cat#CRL-3216 RRID: CVCL_0063
Human: 293T-GP	(Flatz et al., 2010)	N/A
Mouse: RAW 264.7	ATCC	Cat#TIB-71 RRID: CVCL_0493

**Experimental models: Organisms/strains**		

Mouse: C57BL/6	Charles Rivers Laboratories	JAX: 000664
Mouse: FcgRnull/C3KO	This paper	N/A
Mouse: FcgRnull	(Fransen et al., 2018)	N/A
Mouse: C3KO (B6;129S4-*C3*^*tm1Crr*^/J)	(Wessels et al., 1995)	JAX: 003641
Mouse: C1qKO (B6(Cg)-*C1qa*^*tm1d(EUCOMM)Wtsi*^/TennJ)	The Jackson Laboratory (Botto et al., 1998)	JAX: 031675
Mouse: TRIM21ko (C57BL/6-*Trim21*^*tm1Hm*^/J)	Dr. Leo James (James et al., 2007)	JAX: 010724
Mouse: FcRn-/- (B6.129X1-*Fcgrt*^*tm1Dcr*^/DcrJ)	The Jackson Laboratory (Yoshimi et al., 2009)	JAX: 003982

**Software and algorithms**		

GraphPad Prism 9	GraphPad Software	RRID: SCR_002798
FlowJo	Tree Star	RRID: SCR_008520
Gen5	Biotek, USA	RRID:SCR_017317
Developer XD Software	Definiens	RRID: SCR_014283
RADIUS	EMSIS	N/A

**Other**		

Strep-tactin purification columns	IBA Lifesciences	Cat#2-1201-025
Anti-kappa LC resin beads (CaptureSelect)	Thermo	Cat#19135010

### Resource availability

#### Lead contact

Further information and requests for resources and reagents should be directed to and will be fulfilled by the lead contact, Daniel Pinschewer (daniel.pinschewer@unibas.ch).

#### Materials availability

Material transfer agreements with standard academic terms will be established to document reagent sharing by the lead contact’s institution.

### Experimental model and subject details

#### Mice and ethics statement

C57BL/6 mice were bred at the Laboratory Animal Science Center (LASC) of the University of Zurich and were purchased from Charles Rivers Laboratories. FcγRnull/C3KO mice were obtained by crossbreeding FcγRnull mice (deficient in FcγRI as well as FcγRIIB, FcγRIII and FcγRIV) (Fransen et al., 2018)) with C3KO mice (Wessels et al., 1995). C1qKO (JAX ID: b6N(Cg)-C1qa^tm1b(EUCOMM)Wtsi^/3J, (Botto et al., 1998)) and FcRn-/- (Yoshimi et al., 2009) mice were purchased from the Jackson laboratory. TRIM21ko mice (James et al., 2007) were generously provided by Dr. Leo James, University of Cambridge, UK. All mice were on a C57BL/6 background. Experimental groups were sex- and age-matched and animals were typically 8-10 weeks old at the start of an experiment. To reduce the number of animals bred for research purposes, animals of both genders were used. Sample size in the studies were chosen based on long-standing experience in our labs, aiming for group sizes generally revealing biologically significant differences. The groups were neither randomized nor were the experiments conducted in a blinded fashion. All animal experiments were performed under SPF conditions, at the Universities of Geneva and Basel in accordance with the Swiss law for animal protection and with authorization from Cantonal Veterinary Offices of the Cantons of Geneva and Basel, respectively, and at the University of Freiburg with authorization from the Regierungspräsidium Freiburg i.Br.

#### Viruses, viral vectors and cell lines

The baby hamster kidney (BHK) fibroblast cell line BHK-21 (ATCC) was used to grow LCMV. Stably WE GP expressing BHK-21 cells (BHK-21 clone 23) were used to generate rCl13/WE, rCl13/WE^∗^ and rCl13/WE-N119S as well as replication-deficient, GFP expressing LCMV vectors (rCl13ΔGP(WE), rCl13ΔGP(WE^∗^)) from cDNA (Flatz et al., 2006, 2010). To generate high-titer stocks of rCl13ΔGP(WE) and rCl13ΔGP(WE^∗^), the vectors were propagated on stably WE-GP and WE-GP^∗^ expressing HEK-293 (ATCC) cells, respectively. These cell lines were created by stable transfection with a plasmid expressing the respective glycoprotein ORFs (followed by an IRES and the puromycin resistance gene) under control of the human elongation factor-1 alpha promoter (Fischer et al., 2007). The WE^∗^ mutation was introduced by site-directed mutagenesis on the original WE GP expressing plasmid. The mouse macrophage cell line RAW 264.7 (ATCC) was used for viral release inhibition (VRI) assays and flow cytometry neutralization tests (FCNT). The mouse fibroblast cell lines MC57G (ATCC) and NIH/3T3 (ATCC) were used to quantify infectious LCMV titers by immunofocus assays and for LCMV PRNT assays. All cell lines were grown at 37°C in a 5% CO_2_ atmosphere. Cell culture media were selected and supplemented according to ATCC recommendations for the respective cell line. Stably transfected GP-expressing cell lines were maintained in 2 μl/ml puromycin supplemented medium but were kept free of puromycin for rCl13ΔGP batch production. Cell lines were not authenticated owing to their origins from trusted international vendors and repositories. All cell lines were regularly tested mycoplasma-negative.

### Method details

#### Antibody administration and SIGN-R1 blockade

Unless specified otherwise, anti-LCMV antibodies and derived molecules were administered intravenously at a dose of 300 μg three days after LCMV infection. To compensate for the shorter half-life of rKL25 IgG1 IHH in infected C57BL/6 mice or rKL25 IgG1 in infected FcRn-/- mice, repeated administration of antibodies was performed as outlined in Figure S1 to mimic the washout of wildtype antibody in infected animals. To do so, the antibody titers of both groups (WT and IHH) in infected mice were compared for 12h after the antibody injection. The relative loss of IHH antibody as compared to WT antibody was calculated as percentage. This percentage determined the additional dose of IHH administered every 12 hours after an initial dose of 600 μg. For the administration of F(ab')2 fragments, the same method was performed. The respective dosing regimens are shown in Figures S1H and S1K. For *in vivo* blockade of SIGNR-1 (CD209b), C57BL/6 mice were administered a total of 200 μg (100 μg i.p. and 100 μg i.v) of anti-mouse CD209b (BioXCell) 2 days after LCMV challenge. This dose exceeded the amounts which reportedly result in the effective blockade of SIGN-R1 *in vivo* (100 μg i.v. (Kang et al., 2004)).

#### Viruses, viral vectors and infection of mice

LCMV strain clone 13 expressing either the wild-type WE glycoprotein (referred to as rCl13/WE herein), the low-affinity KL25 binder WE^∗^ (referred to as rCl13/WE^∗^), the KL25 escape variant WE-N119S (rCl13/WE-N119S) or the envelope glycoprotein of Junin virus strain XJ13 (rCl13/JUNGP) have been described or were generated from cDNA by reverse genetic techniques as described (Flatz et al., 2006; Penaloza-MacMaster et al., 2015; Sommerstein et al., 2015) and were propagated on BHK-21 cells. rCl13/WE and rCl13.1/WE^∗^ were administered intravenously at a dose of 2x10^6^ PFU unless specified otherwise. rCl13ΔGP vectors were administered at a dose of 10^7^ PFU i.v.

#### Virus titration and neutralization tests

LCM virus and vector stocks and infectious titers in mouse blood were determined by immunofocus assay on MC57G, NIH-3T3 and 293T-GP cells (Battegay et al., 1991; Flatz et al., 2010). In short, serial dilutions of samples were prepared in 200 μl MEM /2% FCS and transferred to 24-well plates, and cells were added. After 2-4 hours of incubation at 37°C, 200 μl of viscous medium (1% methylcellulose, 10% FCS in DMEM) were added. Two days later, the supernatant was discarded by flicking off the plates and 4% paraformaldehyde was added for fixation. Then, cell layers were permeabilized with 1% TritonX100 in PBS. After blocking with 5% FCS in PBS, infectious units were revealed using the VL4 rat-anti-LCMV-NP antibody (Battegay et al., 1991) and secondary horseradish peroxidase (HRP)-conjugated goat-anti-rat-IgG (Jackson), followed by a color reaction (DAB, Sigma). The reaction was stopped by washing the plates with tap water and focus forming units were counted either manually or using a C.T.L. BioSpot counter (Immunospot). For determination of viremia, 50 μl of blood (1 drop) was drawn from infected mice directly into Eppendorf tubes filled with 950 μl BSS-heparin (Na-heparin, Braun, 1IE/ml final concentration) and stored at -80°C until further processing.

To perform plaque reduction neutralization tests (PRNT), serial dilutions of antibodies or serum samples were prepared in MEM medium/2% FCS in a volume of 25 μl inside 96-well plates. Serum samples from infected animals were subsequently UV treated to inactivate potential infectious virus. Then, diluted virus stocks (100 PFU/well) were added and the mixture was incubated for 90 min at 37°C. Subsequently, permissive cells (MC57G or NIH-3T3) were added. Infectious foci were visualized as in immunofocus assays.

In flow cytometry-based neutralization tests (FCNT), serial dilutions of antibody preparations or serially diluted mouse sera were incubated with rCl13ΔGP(WE) vector (3x10^3^ PFU/well) in 96-well plates for 90 min at 37°C. Subsequently, the virus-antibody mixtures were transferred onto 96-well plates pre-seeded with RAW264.7 cells (50-70% confluency). After 90 min incubation at 37°C, the culture supernatant was flicked off and replaced with fresh medium. 24 hours later, the culture medium was discarded and the cells were detached and resuspended using Trypsin-EDTA (0.05%, Gibco) or 0.5mM EDTA. The percentage of GFP^+^ cells was determined by flow cytometry (LSR Fortessa, BD) and analyzed using FlowJo software. The percentage of neutralization was calculated with reference to the mean GFP^+^ cells in several control wells where virus had been incubated with medium only instead of test antibody.

#### Monoclonal antibodies and derived constructs

The neutralizing LCMV-GP1 specific monoclonal antibody-producing hybridoma cell lines KL25, WEN3 and WEN1 has been described (Bruns et al., 1983; Eschli et al., 2007; Fallet et al., 2020; Johnson et al., 2015; Seiler et al., 1998b). The hybridoma-produced antibody KL25 is of the IgG1 isotype, WEN3 and WEN1 are of the IgG2a isotype. For recombinant expression, the cDNAs of the antibodies’ light chain (LC) VJ and heavy chain (HC) VDJ elements were individually subcloned into the CMV-promoter-driven mammalian expression vector pXLG1.2, followed by either Cγ1 or Cγ2a constant domains, which corresponded to the Genbank sequences for mouse IgG1 (J00453.1) and IgG2a (J00470.1), respectively. The D265A or IHH mutations (Baudino et al., 2008; Spiekermann et al., 2002) were introduced into the CH2 and CH2/CH3 domains of the expression cassettes, respectively, by site-directed mutagenesis. Recombinant KL25 monoclonal antibodies (rKL25 mAbs) were finally obtained by transient co-transfection of HC and LC expression plasmids in HEK293 or CHO cells at the Protein Expression Core Facility (PECF) of the Swiss Federal Technical Highschool (EPFL, Lausanne, Switzerland) and at Evitria AG (Zurich, Switzerland). Antibodies were purified on protein G columns using an ÄKTAprime plus (GE Healthcare) followed by PBS dialysis. For production of MonoFab (Figure S6A) and AlbuminFab (Figure S6B) constructs of KL25, WEN3 and WEN1, the respective cDNAs were synthesized by GenScript (USA) and subcloned into the pXLG1.2 expression plasmid. D339K and E356K mutations were introduced in the light chain-linked CH3 domain, and K392D, K409D mutations in the heavy chain-linked CH3 domain (see also Figure S6C), creating an electrostatic steering effect (Gunasekaran et al., 2010), which favors HC-LC heterodimerization and hinders HC-HC or LC-LC homodimerization. The corresponding proteins were obtained as described for the WT antibodies above, and were purified using anti-kappa LC resin beads (CaptureSelect, Thermo), followed by PBS dialysis. These monovalent constructs were further purified and reanalyzed by size exclusion chromatography using either a HiLoad 26/600 Superdex 200 pg column (GE Healthcare) (Figure S6D) and a flow rate of 1 ml/min or a Superdex 200 10/300 GL column (GE Healthcare) (Figure 4B) with a flow rate of 0.7 ml/min to get rid of monomers and multimeric protein aggregates, which was subsequently verified by size exclusion chromatography re-analysis (Figures 4B and S6D). Proteins were further analyzed by SDS-PAGE and Coomassie staining following standard procedures. In brief, 4-15% gradient gels were analyzed using non-reducing conditions, while 12% gels were used for analysis under reducing conditions. KL25 Fab fragments were either generated by recombinant expression or by papain digestion of KL25 mAb (IgG Fab preparation kit, Pierce). F(ab')_2_ fragments were produced by pepsin digestion of rKL25 mAb as follows: antibodies were digested in pepsin digestion buffer (200 μg/ml pepsin, Sigma, in 0.1M NaOAc) for 30 min at 37°C, then neutralized with 2M Tris (pH 9) followed by PBS dialysis. The Junin GP-specific neutralizing mAbs QC03-BF11 and OD01-AA09 were generously provided by the Biodefense & Emerging Infections Research Resources Repository (BEI Resources). The mAb MOPC21 and a recombinantly produced 37.7H antibody (Robinson et al., 2016) in mouse IgG1 format served as isotype controls for cell culture and mouse studies, respectively.

#### ELISA and surface plasmon resonance assays

The soluble GP1-Fc (human Fc) and GP-StreptagII protein constructs have been described (Eschli et al., 2007; Sommerstein et al., 2015) and were produced in HEK293 suspension cells as described for recombinant antibodies. Point mutations corresponding to WE^∗^ and WE-N119S were introduced into the respective pXLG1.2-based expression plasmids by site directed mutagenesis. Unpurified supernatants were used for ELISAs whereas GP-StreptagII proteins were purified for SPR assays using Strep-tactin purification columns (IBA GmbH) according to the manufacturer’s protocol.

GP1-specific antibody responses and passively administered KL25 concentrations in the serum of mice were quantified by ELISA as previously described (Bergthaler et al., 2009). In brief, 96-well high binding ELISA plates (Greiner) were coated with 0.7 μg/ml of goat anti-human IgG Fcγ antibody (Jackson) overnight at 4°C in coating buffer (Na_2_CO_3_ 15mM, NaHCO_3_ 35mM, pH9.6). Then plates were blocked with PBS-T (0.05% Tween) with 5% milk powder for 2 hours at RT. PBS-T/milk was used as diluent for Fc-GP1 as well as for the test samples, standard dilutions and detection antibody. After a blocking step, the plates were incubated with GP1-Fc for 1h at RT. After this step and after each of the following steps, the plates were washed 3 times with PBS-T. After the wash, samples and standard dilutions were added and incubated for 1h at RT, using KL25 mAb as a reference standard. If serum antibody titers were measured, samples were diluted 1:5 in PBS-T milk followed by 1:3 serial dilution preparations. For detection, we used a goat anti-mouse IgG HRP conjugate (1:2500, Jackson). In assays detecting monovalent antibody analogues (MonoFab, AlbuminFab), light chain specific goat anti-mouse IgG (Jackson) was used as detection antibody. HRP activity was detected by an ABTS color reaction (Pierce) and plates were read on an ELISA reader at 405nm. For quantification, 4-parameter logistic curve fitting was performed using Gen5 software (Biotek, USA) with KL25 serving as standard.

GP-StreptagII ELISA was performed to measure antibodies specific for the full-length extracellular domain of the LCMV GP1/GP2 (GP-C) complex. 96-well high binding ELISA plates were coated with strep-tactin (IBA) overnight at 4°C. Then, recombinant streptagII-GP protein (corresponding to the non-cleaved extracellular GP1-GP2 domain) was added. Plates were blocked with PBS-T 0.2 % bovine serum albumin (BSA, Sigma) for 2h at RT. The following steps were performed with 1h incubations at RT. GP-StreptagII protein, test samples and detection antibody were diluted in binding buffer (25mM TrisHCI, 2mM EDTA, 140mM NaCl, pH7.6). After washing, samples and standard dilutions were added. KL25 mAb was used as a standard. Goat anti-mouse IgG HRP conjugate (Jackson) was used as detection antibody and bound secondary antibody was visualized with ABTS as substrate (Pierce). SPR assays were performed as previously described (Sommerstein et al., 2015).

FcγR binding ELISA was performed to compare the affinity of antibodies and derived molecules to mFcγRIIb and mFcγRIII. 96-well high-binding ELISA plates were coated with 2μg/ml Streptavidin (IBA) in coating buffer overnight at 4°C. Then, plates were blocked with PBS-T containing 1% bovine serum albumin (BSA) for 2h at RT. After this step and each following step the plates were washed 3 times with PBS-T and at the end of the staining procedure plates were washed with ddH_2_O. Then, biotinylated FcγIIB (Sino Biological, 200 ng/ml) or biotinylated FcγIII (Acro Biosystems, 200 ng/ml), diluted in PBS-T supplemented with 0.2% BSA were added for 1h at RT. Subsequently plates were washed, antibody constructs (in serial 1:3 dilutions in PBS-T supplemented with 0.2% BSA) were added for 1h at RT. After washing, goat-anti-mouse IgG-HRP conjugate (Fcγ fragment specific F(ab')_2_ fragments, Jackson ImmunoResearch; diluted 1:5000) was added and incubated for 1h at RT. Bound detection antibodies were visualized with ABTS as substrate and plates were read at 405 nm absorbance (Pierce).

#### Viral release inhibition (VRI) assay

3x10^5^ RAW264.7 cells in 1 ml of culture medium were seeded in individual 24-well cell culture wells. After overnight incubation at 37°C, culture medium was discarded and 10^6^ PFU (multiplicity of infection, ∼MOI=2) of virus in 200 μl was added to each well. After 90 min incubation at 37°C the medium was exchanged to remove the viral inoculum. After additional 3.5 h of incubation at 37°C, the medium was removed again and the cell layer was carefully washed 5 times with warm PBS to eliminate any residual viral inoculum. Then, the culture was overlaid with antibody-containing medium or control medium and was incubated for 7 more hours at 37°C unless specified otherwise. To quantify the released virions, culture supernatants were centrifuged at 2000 g for 5 min to get rid of cell debris followed by RNA extraction using the QIAamp viral RNA mini kit (Qiagen). For VRI assays with IC-21 cells, which exhibiting slower virus production kinetics than RAW264.7 cells, the antibody-containing medium was added 12 hours post infection and supernatant was collected 24 hours post infection.

#### Viral RNA quantification by TaqMan RT-PCR

To collect tissues, mice were lethally anesthetized with pentobarbital (100 μl i.p.) and perfused transcardially with 10 ml ice-cold PBS. 1-2 mm sized organ pieces were directly harvested into tubes containing stainless metal beads and 1 ml of Trizol (Invitrogen) or 650 μl of Qiazol (Qiagen). Tubes were immediately transferred to dry ice and then stored at -80°C for later processing. Frozen samples were thawed and homogenized for 3 min in a TissueLyser II (Qiagen) at 30 Hz. After homogenization, RNA was extracted from Trizol or by the RNeasy 96 Universal Tissue Kit (Qiagen) according to the manufacturers’ instructions. RNA pellets were resuspended in 30 μl DEPC-treated water, vortexed and quantified in a NanoDrop 2000 device (Thermo Scientific). RNA concentrations of samples were standardized for RT-qPCR assays. For viral RNA extraction from mouse serum and cell culture supernatant, the QIAamp viral RNA mini kit (Qiagen) was used and a standard volume of eluate was processed for RT-qPCR. To extract total RNA from RAW264.7 cells, 1ml Trizol was directly added into each 24-well of the culture plates. LCMV nucleoprotein-specific TaqMan RT-PCR was performed as previously described (Pinschewer et al., 2010). In viral co-infection experiments, TaqMan RT-PCR was conducted as described in (Johnson et al., 2015) to individually quantify each one of the co-infecting viruses by means of primer/probe sets targeting a stretch of viral RNA that was engineered to carry a non-coding nucleotide tag. This method discriminates reliably between the two viruses and is highly accurate as validated for a range of 10^2^ to 10^8^ genome copies per reaction (PCR performance parameters: R^2^ of >0.99; Slope of -3.4 and -3.6, respectively (Johnson et al., 2015)). In organs, absolute viral RNA copies were calculated per 100 ng of cellular RNA whereas viral RNA copies in serum were back-calculated and expressed as absolute copies per ml of serum. *In vitro* RNA transcripts were used as standard.

#### Immunohistochemistry

Mouse tissues were fixed in cold 4% paraformaldehyde overnight and then embedded in paraffin. Sections were processed for immunohistochemistry as follows. Tissue sections were first incubated in PBS containing 3% hydrogen peroxide to inactivate endogenous peroxidases. Then, sections were blocked with PBS containing 10% FCS to reduce unspecific binding. The sections were then incubated with primary anti-LCMV nucleoprotein sera as described previously (Bergthaler et al., 2007). Bound primary antibody was stained with biotinylated secondary anti-rat antibody (Vectorlabs) and detected with streptavidin peroxidase (DakiCytomation). Bound secondary antibody was revealed with 3.3’-diaminobenzidine as chromogen (DakoCytomation). Hemalum (Merck) was used to counterstain nuclei. Slides were scanned by using a MIRAX Midi slide scanner (ZEISS, Germany) at 200X magnification.

#### Transmission electron microscopy (TEM)

Monolayers of RAW264.7 cells were washed 3 times with RT PBS. Then, the fixative solution (2.5% Glutaraldehyde (Sigma) and 2% PFA (Fischer) in 0.1M sodium cacodylate (Sigma) buffer) was added onto the cell layer. After 45 min incubation at RT, the cells were scrapped by means of a cell scraper, were pelleted by centrifugation and the supernatant was discarded. The cells were then suspended in fixative buffer and incubated for another 30 min. Then, cells were washed with 0.1M sodium cacodylate buffer three times, with each washing step consisting of 10 minutes incubation on ice followed by centrifugation. The fixed samples were subsequently embedded in low melting agarose and upon solidification, the blocks were trimmed into 1-2 mm cubes which were washed three times with PBS. Agarose cubes were post-fixed in 1% buffered Osmium Tetroxide (Electron Microscopy Sciences) for 1h at 4°C and rinsed with distilled water. Then, en-bloc staining was performed by incubation in aqueous Uranyl Acetate for 1h at 4°C in the dark. The cubes were then dehydrated by series of ethanol concentrations in distilled water. Dehydrated samples were washed in acetone (Electron Microscopy Sciences) and finally embedded in a mixture of resin/acetone first and then in pure Epon812 resin (Electron Microscopy Sciences). Embedding was carried out in a 60°C oven for 48h until the epoxy resin had hardened to enable sectioning. Semi-thin sections were cut from blocks with a glass knife and the blocks were selected for thinning. Thin sections were cut with diamond knives and placed on copper grids and impregnated with uranyl acetate and lead citrate. A transmission electron microscope operating at 80kV (FEI Tecnai G2 Spirit TEM) was used for imaging. Images were recorded using a EMSIS Veleta camera (operated by RADIUS software from EMSIS).

#### TEM immunogold staining

For TEM Immunogold staining a pre-embedding method was used (Pinschewer et al., 2004). All steps were performed at RT. 12h after VRI assay monolayers of RAW 264.7 cells were fixed *in situ* for 15 min with fixative solution (0.1% Glutaraldehyde, 3% PFA in 0.1M cacodylate buffer), brought into suspension by a cell scrapper and further fixed for another 15 min in the same fixative solution. The cells were washed three times with PBS and then quenched for 10 min with blocking solution (50mM glycine, 0.1%BSAc (Aurion Immunogold) in PBS). After an additional washing step, the cells were incubated with 20 μg/ml rKL25 IgG2a in PBS (0.05% BSAc) for 2 hours. The cells were washed three more times and incubated for 2 hours with goat anti-mouse immunoglobulin (IgG and IgM, heavy and light chains) conjugated to 10nm gold particles (BBInternational). Then cells were washed twice with PBS and once with 0.1M cacodylate buffer. Fixed samples were embedded in agarose and further processed as for regular TEM.

### Quantification and statistical analysis

#### Quantification of immunohistochemistry

To assess the density of LCMV virus- and vector- infected cells in organs, an automated analysis of LCMV-NP staining was performed using a customized ruleset for Definiens cognition network technology® (Definiens, Munich) on entire sections of organs captured by slide scanner. The regions to analyze were predefined manually for each organ of interest. Subsequently, RGB color layer values were used to detect the DAB signal within each region of interest (ROI). The total percentage of LCMV-infected tissue was calculated from the ratio of total DAB-stained surface to total ROI.

#### Statistical analysis

The GraphPad Prism software (v9, GraphPad Software, San Diego, California) was used for all statistical analyses. When two groups were compared, statistical significance was assessed by two-tailed unpaired or paired Student’s *t* tests, whereas single measurement comparisons in more than two groups were assessed by one-way ANOVA followed by Tukey’s post-tests for multiple comparisons. For several comparisons to a single reference group, Dunnett’s post-tests were used. Viral load and viral RNA data were log-converted to obtain a near-normal distribution prior to statistical analysis. *P*-values <0.05 were considered statistically significant (indicated as ^∗^ in figures), and *p*<0.01 was considered highly significant (indicated as ^∗∗^ in figures). *p*>0.05 was considered not statistically significant (“ns”). The number of experimental animals “*n*” per group, the type of error bar displayed and the tests performed for statistical analysis are indicated in each figure legend.

## Data Availability

• Raw data of the experimental results reported in this study have been deposited with Zenodo and are publicly available as of the date of publication. The DOI is listed in the key resources table. • This paper does not report original code. • Any additional information required to reanalyze the data reported in this paper is available from the lead contact upon request.

## Supplemental information

Document S1. Figures S1–S6 and Table S1

Document S2. Article plus supplemental information
