# Supplementary material for: Antibody bivalency improves antiviral efficacy by inhibiting virion release independently of Fc gamma receptors
Source: Cell Rep. 2022 Feb 1;38(5):110303. doi: 10.1016/j.celrep.2022.110303 (PMC8822495; doi:10.1016/j.celrep.2022.110303)
Supplement: Document S1. Figures S1–S6 and Table S1 [file mmc1.pdf]

Cell Reports, Volume *38*

Supplemental information

Antibody bivalency improves antiviral efficacy by inhibiting virion release independently of Fc gamma receptors

Mehmet Sahin, Melissa M. Remy, Benedict Fallet, Rami Sommerstein, Marianna Florova, Anna Langner, Katja Klausz, Tobias Straub, Mario Kreutzfeldt, Ingrid Wagner, Cinzia T. Schmidt, Pauline Malinge, Giovanni Magistrelli, Shozo Izui, Hanspeter Pircher, J. Sjef Verbeek, Doron Merkler, Matthias Peipp, and Daniel D. Pinschewer

Figure S1

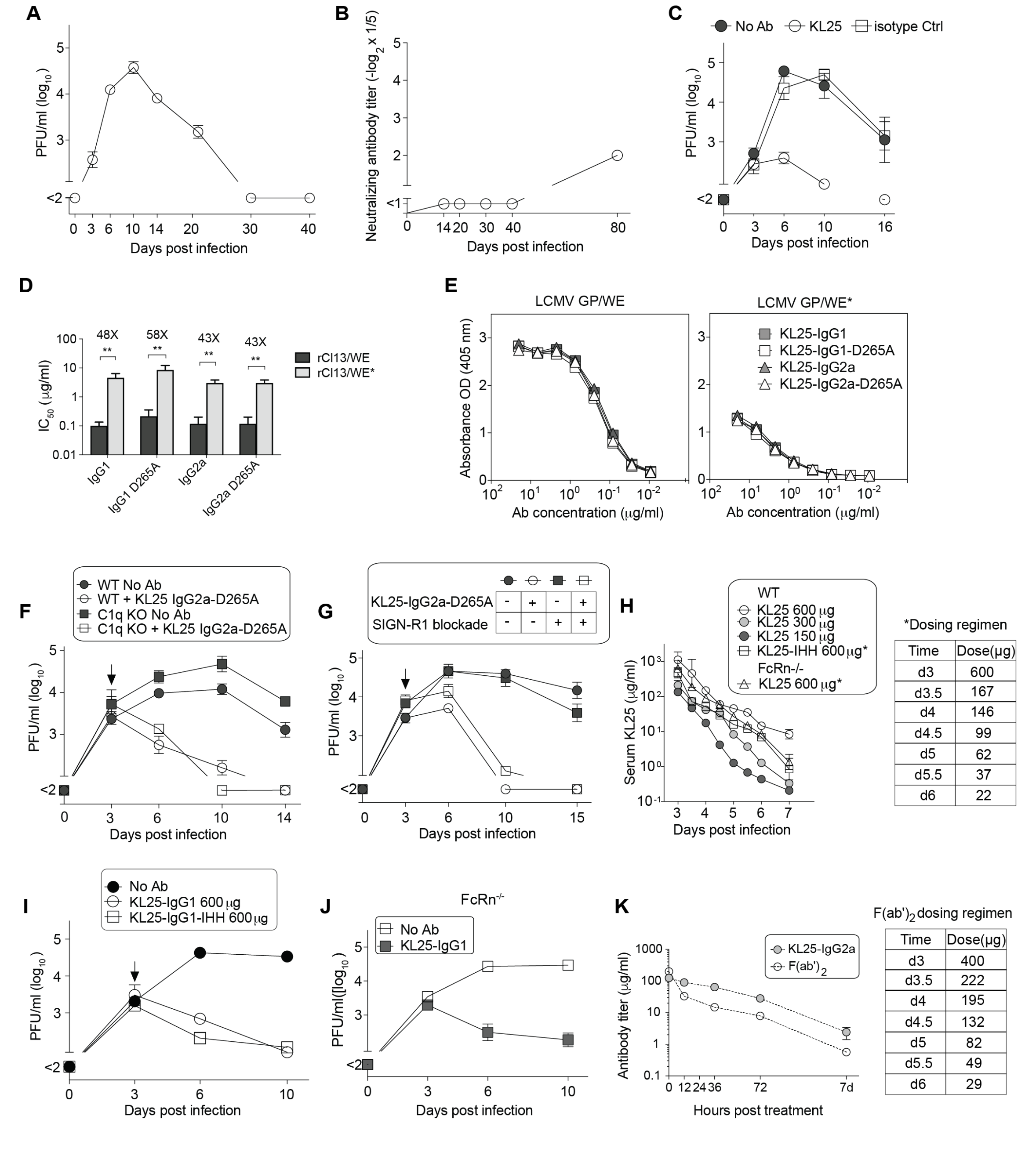

*Dosing regimen

Time	Dose(μg)
d3	600
d3.5	167
d4	146
d4.5	99
d5	62
d5.5	37
d6	22

$F(ab')_2$ dosing regimen

Time	Dose(μg)
d3	400
d3.5	222
d4	195
d4.5	132
d5	82
d5.5	49
d6	29

Figure S1. The course of rCl13/WE infection in WT mice, characterization of KL25 isotype and Fc variants and KL25-mediated protection independently of C1q, SIGN-R1 or FcRn. Related to Figure 1.

(A, B) We infected WT mice (n=4) with rCl13/WE on day 0 and determined viremia (A) and nAb titers by PRNT (B) over time. (C) We infected WT mice with rCl13/WE, treated them with KL25 or isotype control antibody on day 3 or left them untreated ("no Ab"), and followed viremia over time. (D) PRNT assays against rCl13/WE or rCl13/WE* were conducted to determine the half maximal inhibitory concentration (IC_{50}) of KL25 when expressed as IgG1 or IgG2, with either a WT Fc domain or the D265A mutation. Bars indicate mean ±SEM of 3

values (IgG1) or 2 values (IgG2a) obtained from 2 independent experiments. **$p<0.01$ by unpaired Student's t test. (E) WE- and WE*-specific binding were determined by ELISA. Symbols indicate single values from one representative experiment out of two. (F) We infected WT and C1q KO mice with rCl13/WE*, treated them with KL25 on day 3 or left them untreated ("no Ab"), and followed viremia over time. (G) WT mice were given SIGN-R1-blocking antibody or no blocking antibody and all animals were infected with rCl13/WE*, then half the animals in each group were administered KL25-D265A on day 3 (see chart) and viremia was monitored. (H) WT (n=4) and FcRn-/- mice (n=4) were infected with rCl13/WE and treated with titrated doses of KL25 or of KL25-IHH, either as a single dose (KL25 in WT mice) or in a repeated dosing regimen as outlined in the chart (KL25-IHH in WT mice; KL25 in FcRn-/- mice), aimed at mimicking the washout of KL25 in WT mice. KL25 and KL25-IHH concentrations were determined over time in serum by ELISA. (I) Viremia was monitored in WT mice treated with KL25 or KL25-IHH as outlined in (H). (J) Viremia was also determined in FcRn-/- mice repeatedly dosed with KL25 as described in (H). (K) Serum KL25 concentrations in mice (n=4) from the experiment in Fig 1G were determined by ELISA. KL25 was given as a single dose of 300 μg on day 3, whereas $F(ab')_2$ fragments were administered every 12 hours as outlined in the chart. Symbols indicate the mean±SEM of 3 (KL25 treatment) to 4 mice per group ($F(ab')_2$-treatment). One representative experiment of or two (A-E, K) is shown. Symbols in panels (A-C, F-J) show the mean ±SEM of 4 mice per group.

Figure S2

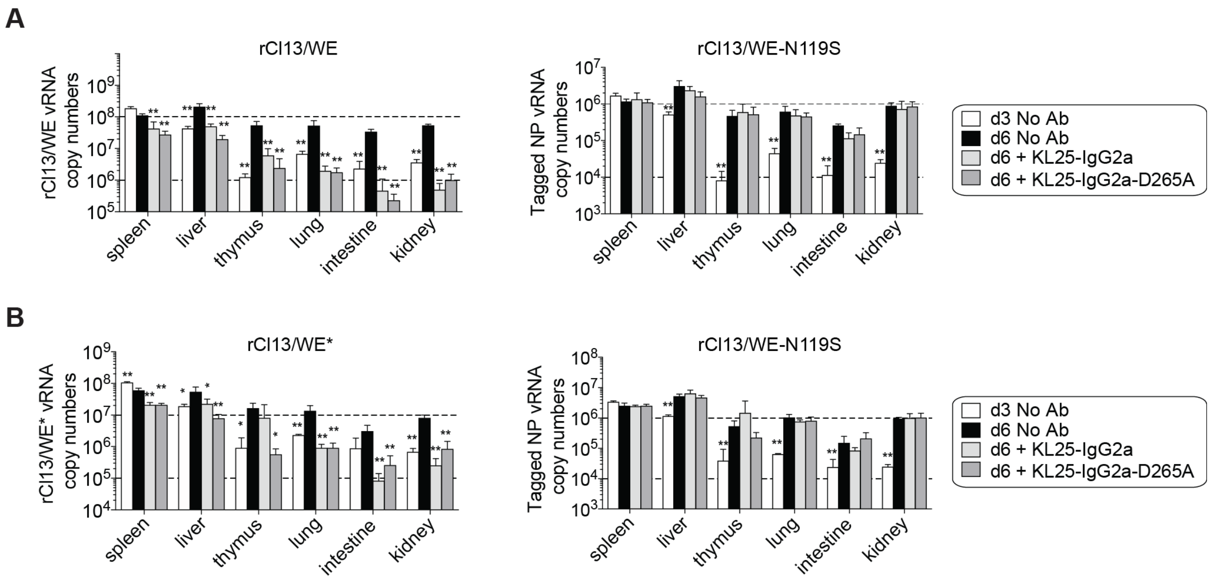

Figure S2. KL25 and KL25-D265A effects on rCl13/WE and rCl13/WE* dissemination in organs don't alter viral loads of a co-infecting virus that escapes antibody binding. Related to Figure 2.

WT mice (n=4) were coinfected with rCl13/WE and rCl13/WE-N119S (A) or rCl13/WE* and rCl13/WE-N119S (B) at a dose of 10^6 PFU each on d0. On day 3 the animals were treated with 300 μg of KL25-IgG2a, 300 μg of KL25-IgG2a-D265A or were left untreated ("no Ab"). The indicated organs were harvested on d3 and on d6 for RNA extraction. Total RNA copies of each co-infecting virus were quantified by TaqMan RT-qPCR. A non-coding genetic tag in the TaqMan-targeted NP sequence of rCl13/WE-N119S allowed us to discriminate its RNA copy numbers from those of the respective co-infecting virus and to individually quantify both co-infecting viruses in the same sample. *$p<0.05$, **$p<0.01$ as determined by one-way ANOVA and Dunnett's post-test comparing the d6 "no Ab" group against the other groups. Bars represent the mean±SEM. Representative results from two independent experiments are shown.

Figure S3

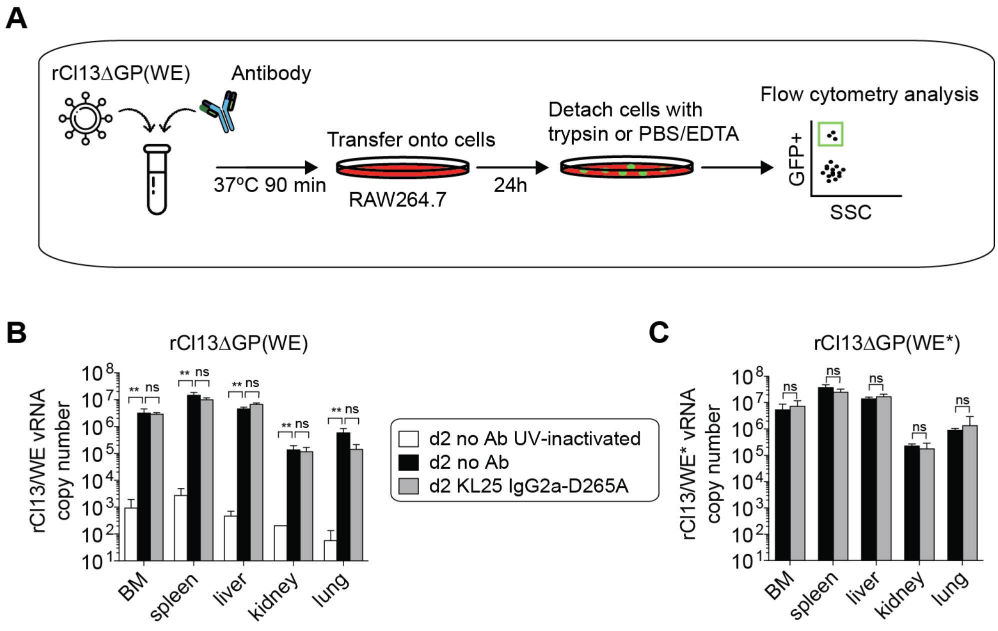

Figure S3. KL25 effect on LCMVΔGP single-round vector entry into organs. Related to Figure 2.

(A) Schematic of the flow cytometry-based neutralization test (FCNT). rCl13ΔGP(WE) vector particles were incubated with either test antibody or control at 37°C for 90 min in a test tube, then the mixture was added to RAW264.7 cells, followed by 24h of culture. Subsequently the cells were detached and the percentage of GFP-expressing (rCL13ΔGP(WE)-infected) cells was determined by flow cytometry to calculate the percentage of vectors neutralized by the test antibody. (B, C) WT mice were administered 300 μg of KL25 antibody or were left untreated ("no Ab"). Five hours later the animals were inoculated with 10^6 PFU of rCl13ΔGP vectors pseudotyped with either the WE (rCl13ΔGP(WE); B) or WE* GP (rCl13ΔGP(WE*); C). Two days after the challenge the organs were harvested for RNA extraction. Total vector RNA copy numbers were determined by RT-qPCR. UV inactivated vector was used as control and demonstrated that the vector RNA determined in live (not UV-treated) vector-inoculated groups of mice reflected intracellular replication of the non-cytolytic vector. BM: Bone marrow. Bars represent the mean±SD of 3 ("d2 no Ab UV-inactivated") or 4 mice ("d2 no Ab" and "d2 KL25") per group. **$p<0.01$ as determined by one-way ANOVA on log-converted values, followed by Dunnett's post-test to compare the d2 "no Ab" group against the other two groups (B, C). Representative results from two independent experiments are shown.

Figure S4

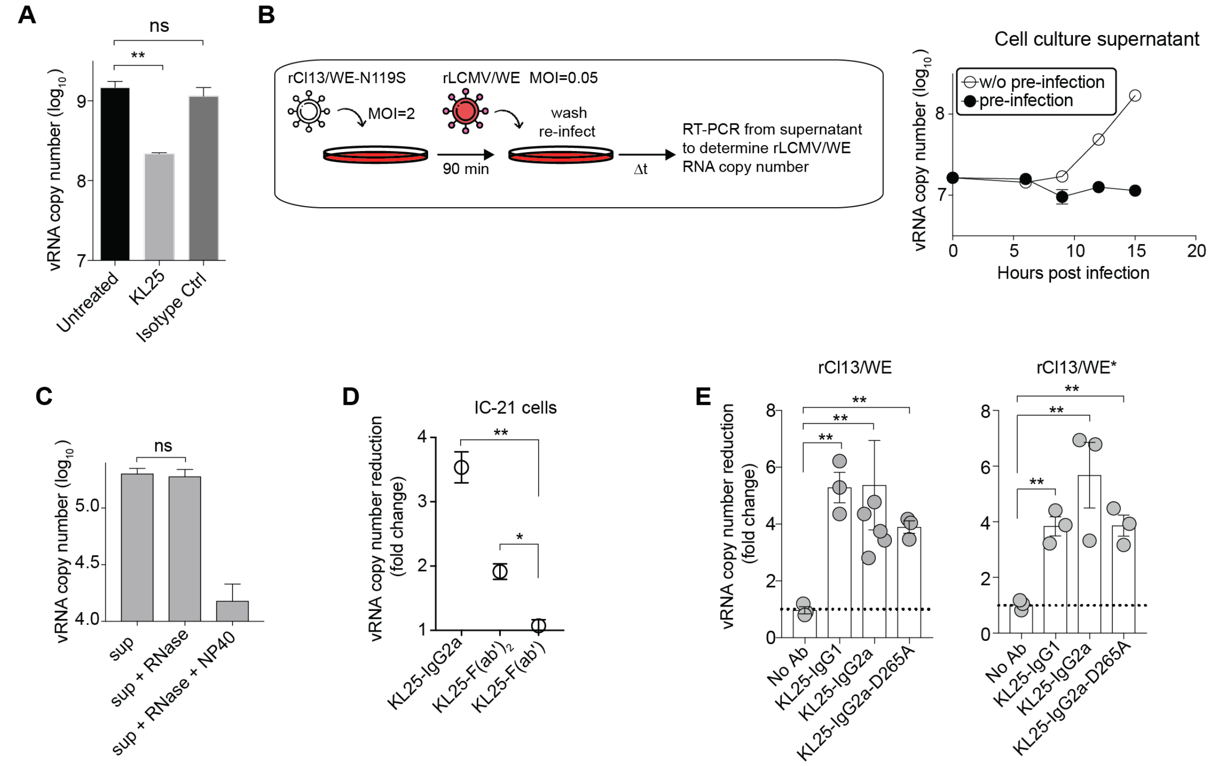

Figure S4. VRI assay activity requires specific antiviral antibody, is not measurably influenced by viral re-infection cycles of cultured cells, detects enveloped cell-free viral RNA, can be conducted on IC-21 macrophage cells and when performed with bivalent antibody works independently of FcγR interactions and antibody isotype. Related to Figure 3.

(A) VRI assay was performed as described in Figure 3A. Infected RAW264.7 cells were treated with KL25-IgG1 or IgG1 isotype control antibody MOPC-21 or were left untreated and cell culture supernatants were collected 12h post infection. Total viral RNA in supernatant was determined by TaqMan RT-qPCR. (B) RAW264.7 cells were infected with rCl13/WE-N119S (MOI = 2; "pre-infection") or left uninfected ("w/o pre-infection). 6 hours later cells were washed and rCl13/WE (MOI = 0.05) was added to each group. Without any further wash step, supernatant samples were collected at the indicated time points. Total viral RNA copies were determined as in (A), confirming that the virus added after six hours was unable to amplify itself, owing to homotypic interference, as expected. This excluded viral re-infection cycles in the VRI assay as a major source of viral RNA in supernatant. (C) A VRI assay was performed with KL25 treatment as in (A). 10h after the infection supernatants were collected and each replicate sample was split into 3 aliquots. These aliquots were incubated with either RNAse alone, RNAse and NP-40 (to disrupt viral membranes) or were left untreated. Two hours later total viral RNA copy numbers were determined. (D) A VRI assay was performed on rCl13/WE-infected IC-21 cells treated

with 10 μg/ml of the indicated KL25 formats or left untreated (no Ab). The antibody efficacy was determined as fold change when dividing the mean RNA copy number of the untreated group by the copy number of individual samples of the treated groups. (E) The VRI activity of KL25-IgG1, KL25-IgG2 and their D265A variants on rCl13/WE- and rCl13/WE*-infected RAW264.7 cells. Symbols represent mean±SEM (B, D) or individual values (E) of 3 technical replicates. Bars show the mean±SEM of 3 technical replicates (A, C, E). *$p<0.05$, **$p<0.01$ as determined by one-way ANOVA with Dunnett's post-test, conducted on log-converted values. Representative results of two independent experiments are shown.

Figure S5

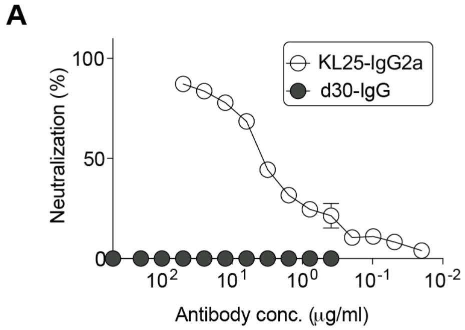

Figure S5. The neutralizing activity of polyclonal d30-IgG. Related to Figure 3.

(A) The neutralizing activity of polyclonal d30-IgG was determined by PRNT. KL25 was included in the experiment as positive control. Symbols show the mean±SEM of 2 technical replicates. Representative results from two independent experiments are shown.

Figure S6

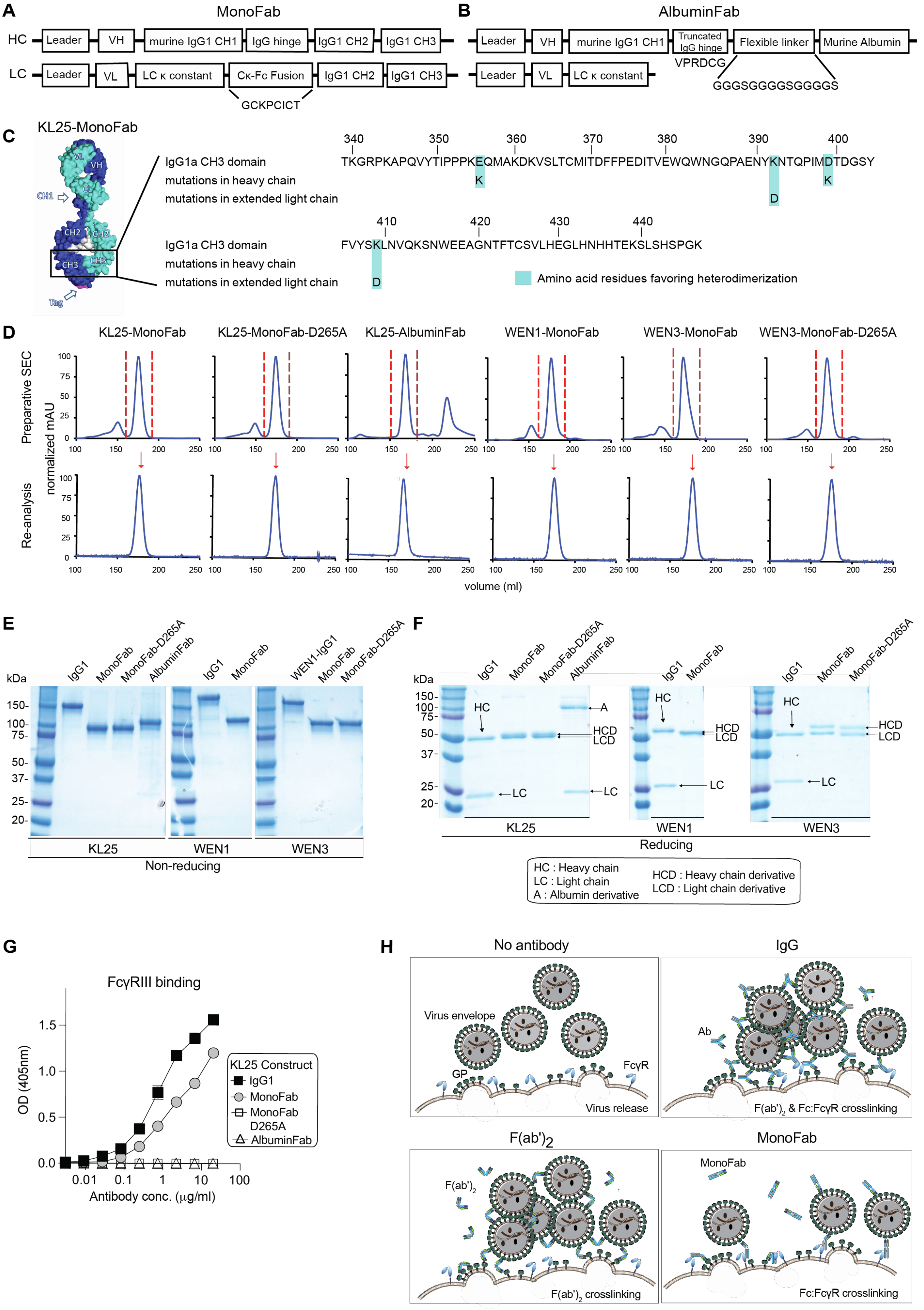

Figure S6. Domain structure of MonoFab and AlbuminFab constructs, Fc mutations introduced to favor MonoFab heterodimerization, purification and validation of MonoFab and AlbuminFab monomers by size

exclusion chromatography, the constructs' analysis by SDS-PAGE and binding to FcγR III, and schematic postulate how antibody FcγR interactions as well as bivalency contribute to tethering of virions to infected cells. Related to Figure 4.

Domain structure of the expression cassettes used for recombinant expression of MonoFab (A) and AlbuminFab (B) from mammalian expression vectors. All immunoglobulin domains were of murine origin. (C) Schematic representation of KL25-MonoFab. The IgG1 CH2 and CH3 domains were fused to the kappa light chain, connected by a part of the hinge domain. Amino acid point mutations as indicated were introduced to favor heterodimerization between the CH3 domains of the heavy and extended light chain CH3 (purple: heavy chain CH3, torques: light chain CH3). (D) After affinity chromatography we performed size exclusion chromatography (SEC) to purify the indicated monovalent antibody constructs (preparative SEC, upper row) and performed re-analysis SEC runs to verify we had recovered a homogenous monomeric, non-aggregated protein for use in cell culture and mouse studies. (E,F) The indicated monovalent antibody constructs and reference IgG1 molecules were analyzed by non-reducing (E) as well as by reducing SDS-PAGE with Coomassie staining (F). Bands corresponding to the respective expected amino acid chains are indicated in (F). (G) Binding of the indicated KL25 constructs to FcγRIII was tested by ELISA (compare also Fig. 4C). (H) Schematic representation of the postulate how antibody bivalency and interactions with cellular FcγRs both contribute to tether virions to infected cells.

Suppl. Tbl. I. Characteristics of WE glycoprotein variants used in this study. Related to Figure 1.

Analyte[1]	GP-C[2]	GP point mutation[3]	KD (M)[4]	SD KD[5]	ka (1/Ms)[6]	SD ka[7]	kd (1/s)[8]	SD kd[9]
KL25 Fab	WE	-	5.04E-09	7-19E-10	6.31E+03	3.18E-05	3.18E-05	2.33E-06
	WE*	N121K	4.00E-08	3.08E-09	6.33E+03	2.53E-04	2.53E-04	7.50E-05
	WE-N119S	N119S	>10E-05 [10]	n.a. [11]	n.a. [11]	n.a. [11]	n.a. [11]	n.a. [11]
WEN3 Fab	WE	-	7.07E-08	7.07E-11	4.24E+03	4.95E+01	3.00E-04	3.54E-06
	WE*	N121K	4.22E-08	2.83E-10	9.49E+03	4.17E+02	4.01E-04	2.05E-05
	WE-N119S	N119S	5.83E-08	1.27E-09	7.06E+03	4.74E+02	4.11E-04	3.68E-05

[1] Surface plasmon resonance (SPR) was performed to test the binding of KL25 Fab fragments to LCMV GP variants

[2] Recombinantly expressed extracellular domains of LCMV GP-C variants were immobilized on SPR chips

[3] Point mutations of the GP-C variants tested

[4] Binding affinity of KL25-Fab – GP-C pairs

[5] Standard deviation of binding affinity of KL25-Fab – GP-C pairs, as determined in duplicate measurements of five to six titrated analyte concentrations (Sommerstein et al., 2015).

[6] On-rate

[7] Standard deviation of on-rate as determined in duplicate measurements of five to six titrated analyte concentrations (Sommerstein et al., 2015).

[8] Off-rate

[9] Standard deviation of off-rate as determined in duplicate measurements of five to six titrated analyte concentrations (Sommerstein et al., 2015).

[10] No binding detectable at 500 nM analyte concentration.

[11] Not applicable.
